# Bioinspired copper single‐atom nanozyme as a superoxide dismutase‐like antioxidant for sepsis treatment

**DOI:** 10.1002/EXP.20210267

**Published:** 2022-07-13

**Authors:** Ji Yang, Ruofei Zhang, Hanqing Zhao, Haifeng Qi, Jingyun Li, Jian‐Feng Li, Xinyao Zhou, Aiqin Wang, Kelong Fan, Xiyun Yan, Tao Zhang

**Affiliations:** ^1^ Collaborative Innovation Center of Chemistry for Energy Materials (iChEM), Dalian Institute of Chemical Physics Chinese Academy of Sciences Dalian China; ^2^ CAS Engineering Laboratory for Nanozyme, Key Laboratory of Protein and Peptide Pharmaceutical, Institute of Biophysics Chinese Academy of Sciences Beijing China; ^3^ Collaborative Innovation Center of Chemistry for Energy Materials (iChEM) College of Chemistry and Chemical Engineering Xiamen University Xiamen China; ^4^ CAS Key Laboratory of Science and Technology on Applied Catalysis Dalian Institute of Chemical Physics Chinese Academy of Sciences Dalian China; ^5^ University of Chinese Academy of Sciences, Chinese Academy of Sciences Beijing China; ^6^ Nanozyme Medical Center, School of Basic Medical Sciences Zhengzhou University Zhengzhou China; ^7^ Key Laboratory of Infection and Immunity Institute of Biophysics Chinese Academy of Sciences Beijing China; ^8^ School of Engineering and Applied Science University of Pennsylvania Philadelphia Pennsylvania USA

**Keywords:** bioinspired, reactive oxygen species, sepsis, single‐atom nanozyme, superoxide dismutase

## Abstract

Sepsis is a systemic inflammatory response syndrome with high morbidity and mortality mediated by infection‐caused oxidative stress. Early antioxidant intervention by removing excessively produced reactive oxygen and nitrogen species (RONS) is beneficial to the prevention and treatment of sepsis. However, traditional antioxidants have failed to improve patient outcomes due to insufficient activity and sustainability. Herein, by mimicking the electronic and structural characteristics of natural Cu‐only superoxide dismutase (SOD5), a single‐atom nanozyme (SAzyme) featuring coordinately unsaturated and atomically dispersed Cu‐N_4_ site was synthesized for effective sepsis treatment. The de novo‐designed Cu‐SAzyme exhibits a superior SOD‐like activity to efficiently eliminate O_2_^•−^, which is the source of multiple RONS, thus blocking the free radical chain reaction and subsequent inflammatory response in the early stage of sepsis. Moreover, the Cu‐SAzyme effectively harnessed systemic inflammation and multi‐organ injuries in sepsis animal models. These findings indicate that the developed Cu‐SAzyme possesses great potential as therapeutic nanomedicines for the treatment of sepsis.

## Graphical abstract

A SOD‐inspired copper single‐atom nanozyme (Cu‐SAzyme) was designed and synthesized by a two‐step scaffold‐adsorption method. The Cu‐SAzyme exhibits high catalytic activity to scavenge superoxide anion, the initiator of ROS, and thus effectively prevents the disease progression in septic animals.

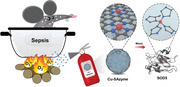

## 1 INTRODUCTION

Sepsis is a severe life‐threatening systemic inflammatory response syndrome caused by microbial infections, leading to extremely high morbidity and mortality worldwide.^[^ ^1^, ^2^ ^]^ During the onset of sepsis, local infection triggers a systemic immune response and overproduces reactive oxygen and nitrogen species (RONS) to damage cells and tissues, which may eventually cause septic shock and multiple organ dysfunction syndrome.^[^ ^3^, ^4^ ^]^ Sepsis is typically featured by acute oxidative damage caused by immune system dysfunction within a short period.^[^ ^5^, ^6^ ^]^ Current mainstream medical therapies focus on suppressing aberrant inflammation and excessive RONS as quickly as possible other than killing the microorganisms.^[^ ^7^, ^8^, ^9^, ^10^, ^11^ ^]^ Particularly, excessive RONS, which is a major cause of multi‐organ failure, has been regarded as a critical target to alleviate the progression of sepsis.^[^ ^12^ ^]^

In sepsis, the activated innate immune system leads to the massive production of pro‐inflammatory cytokines such as tumor necrosis factor (TNF‐α) and IL‐6, causing mitochondrial dysfunction.^[^ ^13^, ^14^ ^]^ Eventually, a large amount of O_2_^•−^ is generated by electron leakage at the ubiquinone site of the mitochondrial respiratory chain.^[^ ^15^, ^16^ ^]^ Under physiological conditions, O_2_^•−^ is eliminated in time by superoxide dismutase (SOD) to maintain redox homeostasis.^[^ ^16^ ^]^ However, overproduced O_2_^•−^ in sepsis far exceeds the scavenging ability of SOD in vivo, which in turn causes oxidative stress and converts into more harmful RONS.^[^ ^12^, ^15^ ^]^ Therefore, exogenous antioxidants are needed to assist in reducing excessive O_2_^•−^ to avoid oxidative damage, especially at the early stage when the systemic bacterial load is partially contained during sepsis. Studies have shown that overexpression of endogenous SOD or administration of exogenous SOD effectively prevents acute organ injury and improves the survival of septic animals.^[^ ^17^, ^18^, ^19^, ^20^, ^21^, ^22^ ^]^ Although natural SOD holds promise for treating sepsis, it is unsatisfactory in practical applications due to its instability under non‐physiological conditions and susceptibility to proteolytic enzymes. In addition, the natural SOD enzymes are typically hard to traverse the cell membrane to scavenge the excessive RONS. Some small molecule SOD mimetics such as M40401 and Tempol have also been reported to partially improve the inflammatory and oxidative symptoms of sepsis, but with low efficacy and side effects.^[^ ^23^, ^24^, ^25^, ^26^ ^]^ Other traditional antioxidants such as hydrogen gas, vitamin C and *N*‐acetylcysteine are also currently available. However, they have been reported to lack efficacy in treating sepsis because of their insufficient activity to diminish complex inflammation and excessive RONS.^[^ ^25^, ^27^, ^28^, ^29^, ^30^ ^]^ Thus, the investigation of useful strategies to effectively curb the outburst of RONS is urgently needed.

Notably, with expeditious development in nanotechnology, various nanomaterials with antioxidant enzyme‐like activities (nanozymes) have emerged in the past decade as promising alternative drugs for the treatment of oxidation‐related inflammatory diseases, including sepsis.^[^ ^31^, ^32^, ^33^, ^34^, ^35^, ^36^, ^37^, ^38^ ^]^ Nanozymes are more stable and less expensive than natural enzymes. Currently, ceria‐zirconia nanozymes and Co‐doped carbon nanozymes (Co/PMCS) have successfully mimicked the antioxidative enzymes such as SOD, catalase, and glutathione peroxidase and exhibited effective therapeutic effects in sepsis.^[^ ^39^, ^40^ ^]^ However, although these nanozymes have been revealed to exhibit multiple antioxidant enzyme activities, their activities are far from the corresponding natural enzymes. Stimulated by the concept of single‐atom catalysis,^[^ ^41^ ^]^ a strategy that has been proven effective in recent years to create highly active nanozymes is to create single‐atom bioinspired nanozymes by simulating the metal coordination structure of metalloenzymes.^[^ ^42^, ^43^ ^]^ Although some single‐atom nanozymes have been successfully developed, the common synthesis method through one‐pot pyrolysis of metal salts, carbon, and nitrogen precursors may weaken the accessibility of the catalytic site by embedding metal atoms in the carbon skeleton and agglomerating atoms.^[^ ^39^, ^44^, ^45^, ^46^, ^47^ ^]^

Herein, a single‐atom nanozyme with a divalent Cu‐N_4_ structure (Cu‐SAzyme) has been de novo‐designed to mimic the electronic and structural features of natural Cu‐only SOD5. Unlike traditional single‐atom nanozyme synthesized by the one‐pot pyrolysis method, the active sites of Cu‐SAzyme obtained via separating carbonization and M‐N coordination are almost evenly exposed on the surface, making them accessible to the substrate and further mediate catalytic reactions. The obtained Cu‐SAzyme showed high SOD‐like activity and good stability at a wide catalytic pH and temperature range. More importantly, Cu‐SAzyme significantly eliminated excess ROS and pro‐inflammatory cytokines in activated inflammatory cells, and effectively reduced multiple organ damage and mortality in septic animals.

## 2 RESULTS AND DISCUSSION

### 2.1 Synthesis and structural characterization

**FIGURE 1:**
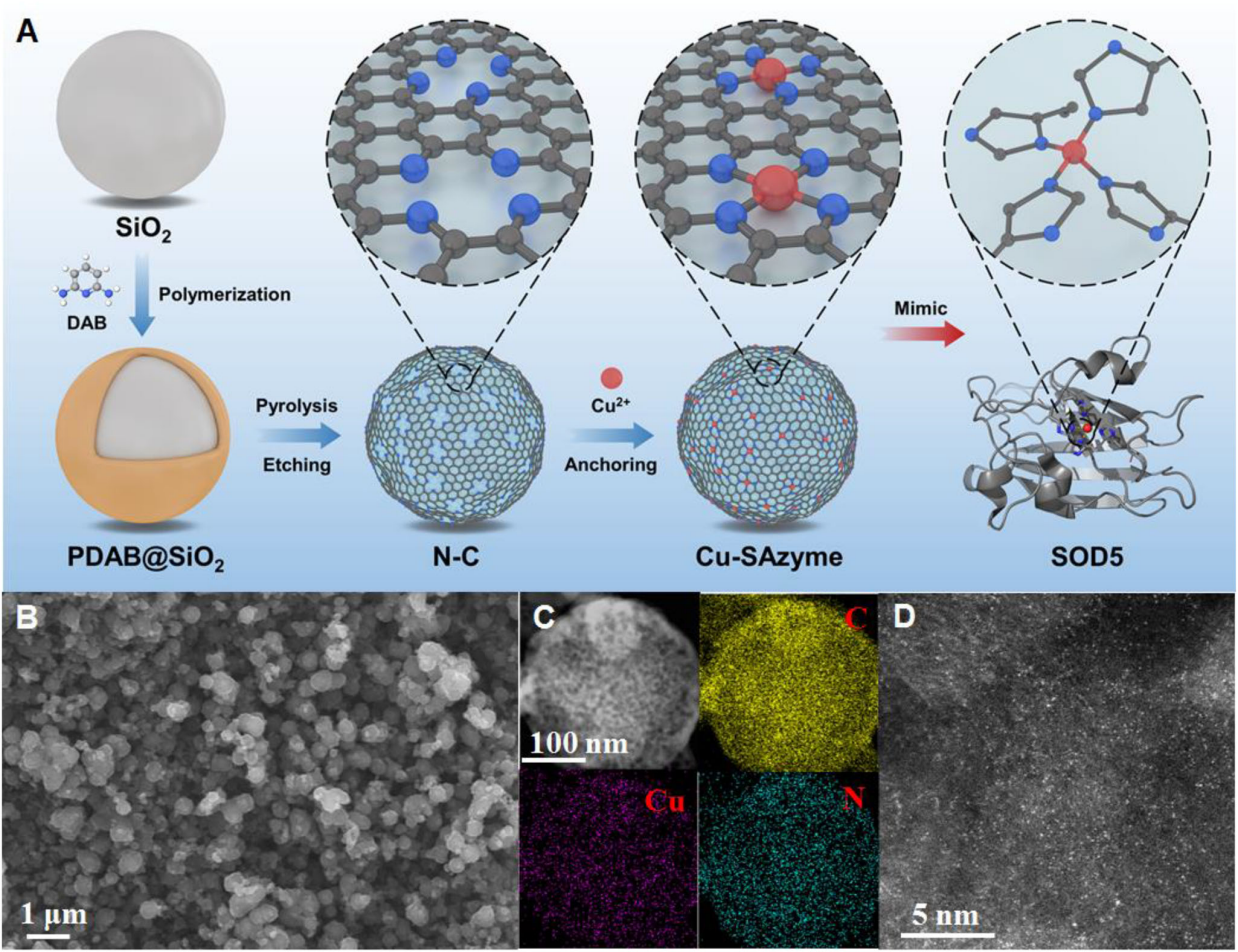
Synthesis schematic illustration and characterization of Cu‐SAzyme. (A) Post‐adsorption strategy to mimic the microenvironment of SOD5. DAB, 2,6‐diaminopyridine. PDAB, polymer of 2,6‐diaminopyridine. (B) SEM image. (C) EDX‐elemental mapping images of C, Cu, N. (D) HAADF‐STEM image

To construct a Cu‐SAzyme, we first synthesized N‐doped carbon (N‐C) support, as we reported earlier.^[^ ^48^ ^]^ In brief, 2,6‐diaminopyridine (DAP) as the N/C source was polymerized on a silica template and pyrolyzed at 800°C for 2 h in an NH_3_/He atmosphere. Afterward, the N‐doped carbon support was obtained by leaching the silica template using HF solution. The obtained N‐C support exhibits the characteristics of low crystallinity graphite structure, large surface area (985 m^2^/g), plentiful mesopores, and abundant pyridinic N species (Figures S1 and S2; Table S1). Then, Cu^2+^ cations were introduced to the N‐C support. Because of the strong binding of pyridinic N to transition metal cations, Cu^2+^ cations were strongly anchored to the pyridinic N sites by simple adsorption, thus producing a well‐defined and uniform micro‐environment (Figure 1A). Compared to those one‐pot pyrolysis methods,^[^ ^49^ ^]^ our approach allowed for the locating of Cu merely on the surface, thus successfully avoiding the embedding of the active sites in the N‐C support and maximizing the accessibility of the metal sites. The Cu loading was 2.0 wt% determined by inductively coupled plasma optical emission spectrometry (ICP‐OES) (Table S2). The XRD patterns of N‐C and Cu‐SAzyme showed merely peaks characteristic of carbon, and no peaks ascribing to Cu or Cu oxide were found, indicating that Cu species were highly dispersed (Figure S1). The scanning electron microscopy (SEM) and transmission electron microscopy (TEM) images in Figure 1B,C showed porous carbon nanoparticles with spherical morphology without any aggregates of metal species, and the energy‐dispersive spectroscopy (EDS) analysis also revealed the uniform dispersion of Cu, C, N elements in the Cu‐SAzyme sample (Figure 1C). The atomic resolution high‐angle annular dark‐field scanning transmission electron microscopy (HAADF‐STEM) clearly showed a high density of single atoms was uniformly dispersed onto the N‐C support (bright dots in Figure 1D and Figure S3). As a control sample, CuNPs/NC was also synthesized by a one‐pot method, which exhibited obvious metal aggregation (Figure S4).

**FIGURE 2:**
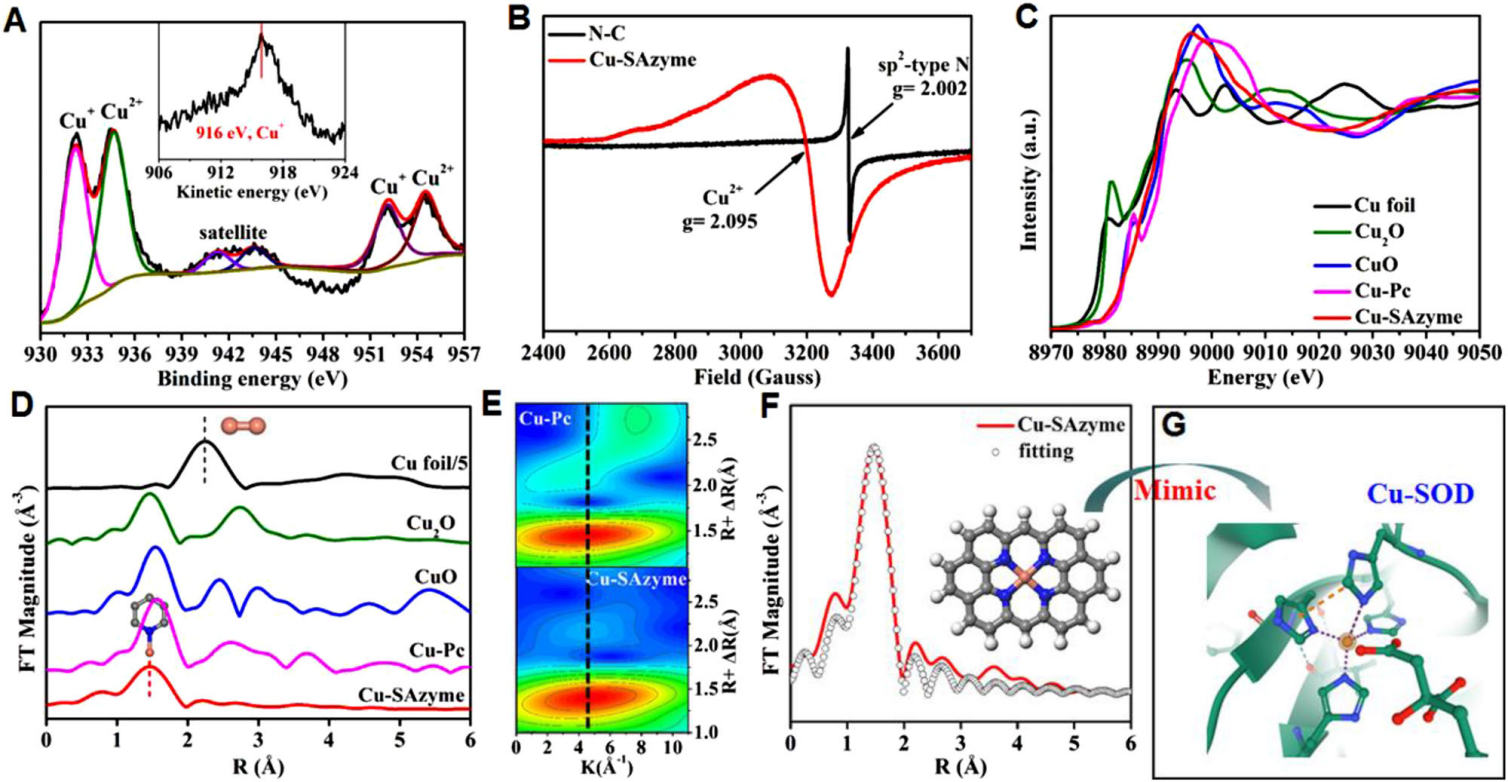
Chemical state and coordination information for Cu‐SAzyme. (A) Cu 2p XPS and LMM Auger spectra. (B) EPR spectra of N‐C and Cu‐SAzyme measured at 100 K. (C) The normalized Cu K‐edge XANES spectra. (D) Fourier‐transform of EXAFS spectra. (E) WT plots of reference Cu‐Pc (top) and Cu‐SAzyme (bottom), respectively. (F) EXAFS fitting curve for Cu‐SAzyme (inset, the Cu‐N_4_ structure). (G) The local Cu‐N_4_ coordination environment in Cu‐only SOD5

To probe into the chemical environments of Cu single atoms in SAzyme, we first performed high resolution X‐ray photoelectron spectroscopy (XPS). As shown in Figure 2A, the XPS spectrum of Cu‐SAzyme exhibited two distinguishable 2p3/2 peaks at 934.6 eV and 932.2 eV, respectively, attributed to Cu^2+^ and Cu^+^ species (Figure 2A).^[^ ^50^, ^51^, ^52^ ^]^ Since the difference in Cu2p binding energy of Cu^0^ and Cu^+^ are merely ca. 0.1 eV, the kinetic energy at 916 eV in LMM Auger spectrum was employed to exclude the presence of metallic species (inset in Figure 2A). Moreover, all Cu atoms were also subjected to electron paramagnetic resonance (EPR) measurements at 100 K to exclude the presence of monovalent Cu species resulting from the Cu^2+^ reduction under ultra‐high vacuum XPS measurements.^[^ ^53^ ^]^ As exhibited in Figure 2B, the new paramagnetic signal in Cu‐SAzyme was clearly presented around *g* = 2.095, clearly indicating that the Cu species were presented in N‐C support as an extremely pure divalent state (Cu^2+^), which possessed the same electronic structure as that of natural SOD5.^[^ ^54^ ^]^ It is noted that the spin resonance peak centered at *g* = 2.002 in N‐C, which is ascribed to the delocalization of the unpaired electrons in sp^2^‐type pyridinic N sites,^[^ ^55^ ^]^ was impressively reduced after post‐anchoring Cu single atoms, confirming the formation of Cu‐pyridinic N interaction in Cu‐SAzyme. This chemical bond of Cu‐pyridinic N could be further identified by the high resolution N1s spectrum in Figure S5A due to the presence of a newly evolving band at 399 eV.^[^ ^56^ ^]^ The interaction between Cu and carbon could be excluded due to the coincident C1s curves between N‐C and Cu‐SAzyme (Figure S5B).

The Cu K‐edge normalized X‐ray absorption near‐edge structure (XANES) spectra have also been investigated to better uncover the detailed structural information in the as‐synthesized Cu‐SAzyme (Figure 2C). In order to more clearly discern the characteristic peak of Cu‐SAzyme, the first‐order derivatives have been conducted and exhibited in Figure S6. The presence of a pre‐edge peak located at 8976.2 eV being assigned to 1s→3d transition in Cu^2+^ was identical to that of the EPR result, indicating the existence of Cu^2+^ in Cu‐SAzyme. In the Fourier‐transformed extended X‐ray absorption fine structure (FT‐EXAFS) spectra, the prominent peak of Cu‐SAzyme located at ca. 1.46 Å was ascribed to the Cu‐N scattering path with the absence of Cu─O (1.55 Å) and Cu─Cu bond (2.3 Å), further indicating the formation of atomically dispersed Cu sites in Cu‐SAzyme (Figure 2D). Correspondingly, the wavelet transforms (WT)‐EXAFS of Cu foil, Cu‐Pc, and Cu‐SAzyme have also been compared and confirmed the absence of Cu─Cu bond in Cu‐SAzyme (Figure 2E bottom, and Figure S5C). Additionally, the maximum WT intensity of Cu‐SAzyme nearing 4.5 Å^–1^ was similar to that of four nitrogen‐coordinated references Cu‐Pc, apparently indicating that the Cu‐N coordination is more likely to present in Cu‐SAzyme (Figure 2E), in concordance with the results of XPS and EPR. The best EXAFS‐fitting analyses clearly showed that the four Cu‐N coordination with an average bond length of 1.96 Å (Figure 2F and Table S3). Evidently, the electronic and structural geometry of Cu‐SAzyme has been identified to be Cu^2+^‐N_4_ coordination, which is analogous to a natural Cu‐SOD enzyme featuring a divalent Cu metal center with four histidine ligands (Figure 2G).^[^ ^54^ ^]^

### 2.2 SOD‐like activity of Cu‐SAzyme nanozyme

**FIGURE 3:**
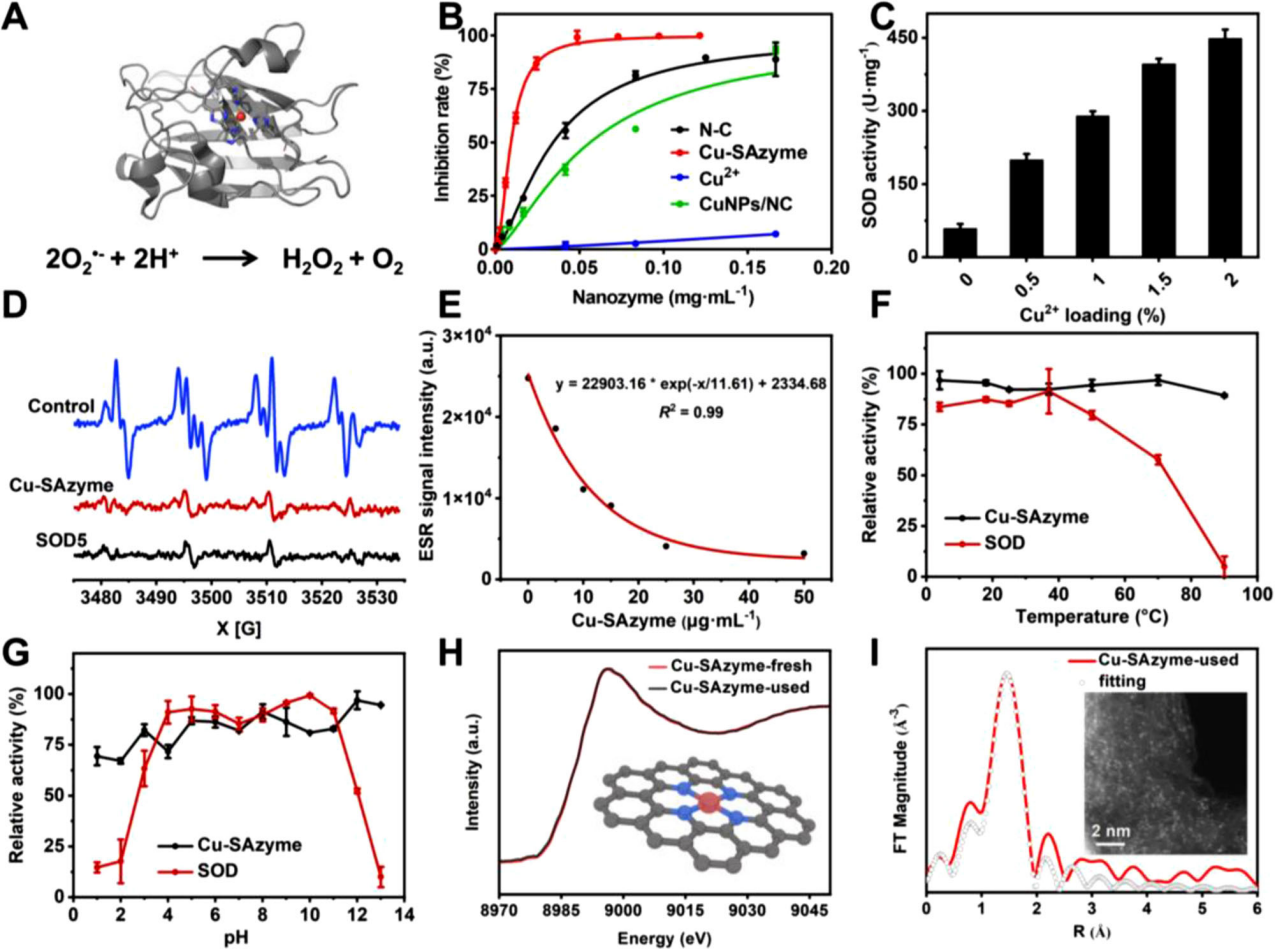
SOD‐like activity of Cu‐SAzyme. (A) Schematic presentation of the Cu‐SOD that scavenges O_2_^•−^ by catalyzing its dismutation reaction. (B) Inhibition rate curve of N‐C and Cu‐SAzyme samples. (C) SOD‐like activity of Cu‐SAzyme samples with different Cu^2+^ loading. (D) Change in DMPO/•OOH ESR signal upon addition of Cu‐SAzyme in the O_2_^•−^ generating system. Control group: 200 mM DMPO, 0.6 mM xanthine and 0.06 U/ml xanthine oxidase (XOD). [Cu‐SAzyme] = 50 µg/ml. [SOD5] = 50 µg/ml. (E) The effect of Cu‐SAzyme concentration on DMPO/•OOH ESR signal. (F) The effect of temperature on SOD‐like activity of Cu‐SAzyme. (G) The effect of pH on SOD‐like activity of Cu‐SAzyme. (H) The comparison of XANES spectra between fresh and usedCu‐SAzyme, inset is the corresponding catalytic process. (I) The Cu‐K edge EXAFS fitting analysis of Cu‐SAzyme‐used in R space, inset is the HAADF‐STEM image

SOD is well known to catalyze the dismutation of O_2_^•−^ into O_2_ and H_2_O_2_ (Figure 3A), which is very important for antioxidant defense in cells. The SOD‐like activity of Cu‐SAzyme was evaluated using a total SOD kit method, in which the reduction of WST‐1 to formazan by O_2_^•−^ produced via the xanthine/xanthine oxidase (Xan/XOD) system was tracked at 450 nm. The excessive free ions in the detection system were chelated by EDTA. The addition of SOD (or nanozyme) to the system dismutates O_2_^•−^, leading to a decrease in formazan production. As shown in Figure 3B, with the increase of Cu‐SAzyme concentration, the inhibition of formazan formation increased significantly, indicating that the Cu‐SAzyme possesses SOD‐like activity under physiologically relevant conditions. In contrast, the SOD‐like activity of N‐C was much lower, and the SOD‐like activity of free Cu^2+^ was negligible. The catalytic activity of CuNPs/NC was even lower than that of N‐C, indicating that no competent active sites were formed in the one‐pot synthesis. Moreover, with the increase of Cu loading from 0.5% to 2%, the SOD‐like activity of Cu‐SAzyme increased from 58.89 to 448.72 U/mg (Figure 3C). The samples synthesized by replacing Cu with other metals also exhibited different SOD‐like activities in the order: Cu > Co > Mn > Zn >Ni (Figure S7). These results indicate that Cu is the key to the high catalytic activity of Cu‐SAzyme.

To further verify the O_2_^•−^ scavenging ability of Cu‐SAzyme, the Xan/XOD system and DMPO were selected as the generator and detector of O_2_^•−^, respectively. DMPO is a spin‐trapping agent that forms a covalent adduct with O_2_^•−^, which quickly converts into an adduct of DMPO/•OOH in an aqueous solution, thus making O_2_^•−^ detectable in the EPR spectrum.^[^ ^57^ ^]^ The characteristic EPR spectrum of the spin adduct DMPO/•OOH exhibited four spectral lines with the relative intensity of 1:1:1:1 and two hyperfine splitting parameters (Figure 3D). The addition of SOD led to a significant decrease in ESR signal intensity due to the efficient dismutation of O_2_^•−^. A similar phenomenon was detected when Cu‐SAzyme was added, suggesting that the Cu center possesses SOD‐like activity. Moreover, the ESR signal intensity of DMPO/•OOH gradually decreased with the increase of Cu‐SAzyme concentration (Figure 3E), indicating that Cu‐SAzyme scavenged O_2_^•−^ in a concentration‐dependent manner. Intriguingly, Cu‐SAzyme exerted SOD‐like activity in temperature‐ and pH‐independent manners (Figure 3F,G). In the environmental range of 4–90°C and pH 1–13, the SOD‐like activity of Cu‐SAzyme remained almost constant, while the activity of natural SOD decreased sharply or even lost under the conditions of high temperature, strong acid, or strong alkali. Overall, our results demonstrated that Cu‐SAzyme could function as a highly active SOD‐mimic.

As exhibited in Figure 3H, the XANES profile of spent Cu‐SAzyme is identical to that of the fresh sample, indicating a better electronic stability after performing the catalytic process (inset in Figure 3H). The FT‐EXAFS fitting analysis clearly showed that the used Cu‐SAzyme (underwent one cycle of catalysis) maintained a Cu‐N_4_ coordination configuration (Figure 3I and Table S3). The STEM images also showed that the used Cu‐SAzyme presented the original spherical shape without any aggregates of metal species (Figure S8A,B). The EDS analysis showed that Cu, C, and N elements were still uniformly dispersed in the used Cu‐SAzyme sample (Figure S8C–E). The atomic resolution HAADF‐STEM also clearly demonstrated that high‐density single atoms were uniformly dispersed on the surface of the used Cu‐SAzyme (inset in Figure 3I). Moreover, the catalytic activity of Cu‐SAzyme remained almost unchanged during storage in an aqueous solution for 3 months (Figure S9), suggesting its good catalytic stability.

### 2.3 Cellular evaluation of antioxidant and anti‐inflammatory properties of Cu‐SAzyme

The SOD‐like activity of Cu‐SAzyme is superior to most reported nanozymes in terms of specific activity per mass of catalyst (Table S4). Encouraged by the high SOD‐like activity, in vitro experiments were conducted to evaluate the antioxidant and anti‐inflammatory capabilities of Cu‐SAzyme. First, to make Cu‐SAzyme exhibit sufficient biocompatibility and dispersity, polyethylene glycol (PEG) was modified on the surface of Cu‐SAzyme. The Fourier transform near‐infrared (FT‐IR) spectrum of the modified Cu‐SAzyme presented the characteristic peaks of PEG groups such as ─CH_2_─, ─C─O─C─, and ─CH_2_O─ (Figure S10). Moreover, the zeta potential of Cu‐SAzyme in water decreased from +41.52 to −3.51 mV after modification (Figure S11), further confirming the successful modification of PEG on the surface. The average size of the PEG‐modified Cu‐SAzyme in H_2_O, PBS, or DMEM medium remained constant (Figure S12), indicating that PEG‐modification was beneficial for particle stability. The SOD‐like activity of the modified PEG‐Cu‐SAzyme was determined to be 430.98 U/mg, which was slightly lower than that of the unmodified Cu‐SAzyme (448.22 U/mg). The difference was not significant (Figure S13), indicating that PEG modification had little effect on the activity of Cu‐SAzyme.

**FIGURE 4:**
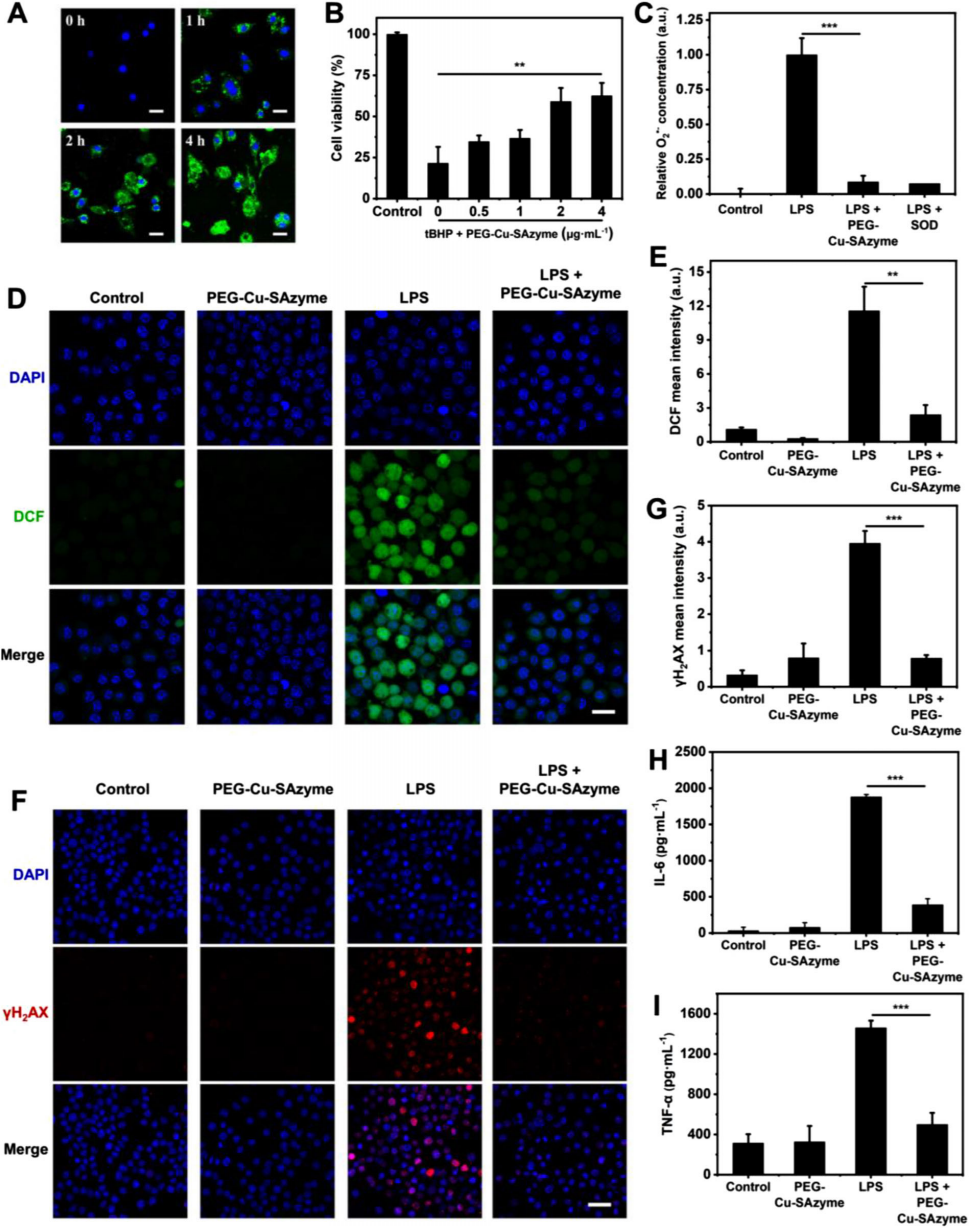
Antioxidant and anti‐inflammatory ability of PEG‐modified Cu‐SAzyme nanozymes in cells. (A) Fluorescence microscope images of Raw264.7 cells incubated with FITC‐labeled PEG‐Cu‐SAzyme within 0–4 h. DAPI, blue; FITC‐PEG‐Cu‐SAzyme, green. Scale bar, 20 µm. (B) Viability of Raw264.7 cells incubated with different concentrations of PEG‐Cu‐SAzyme with or without tBHP stimulation. (C) The concentration of O_2_^•–^ in U937 cells incubated with PEG‐Cu‐SAzyme nanozymes under LPS stimulation. (D) DCF fluorescence signal of Raw264.7 cells under different treatments for measuring ROS levels. DAPI, blue; DCF, green. Scale bar, 50 µm. (E) Quantitative data of DCF fluorescence intensity in (D). (F) γ‐H_2_AX foci immunofluorescence staining of Raw264.7 cells under different treatments for evaluating DNA damage. DAPI, blue; Cy5 labeled secondary antibody, green. Scale bar, 100 µm. (G) Quantitative data of γ‐H_2_AX foci immunofluorescence intensity in (F). (H) The level of cytokine IL‐6 in the culture supernatant of LPS‐stimulated Raw264.7 cells under PEG‐Cu‐SAzyme treatment. (I) The level of cytokine TNF‐α in the culture supernatant of LPS‐stimulated Raw264.7 cells under PEG‐Cu‐SAzyme treatment

The mononuclear phagocyte system activated by infectious factors is one of the main effectors of inflammation and oxidative stress in sepsis.^[^ ^58^, ^59^ ^]^ Therefore, the murine macrophage Raw264.7 cell was selected for follow‐up in vitro experiments, which is one of the most commonly used cell models for inflammation research including sepsis.^[^ ^60^, ^61^ ^]^ Then, we verified the cytotoxicity and endocytosis efficiency of PEG‐Cu‐SAzyme against macrophages. Fluorescein isothiocyanate (FITC) labeled PEG‐Cu‐SAzymes were effectively endocytosed by Raw264.7 cells after 1 h of incubation and were significantly enriched in the cells within 4 h (Figure 4A, Figure S14). Moreover, PEG‐Cu‐SAzyme treatments showed no cytotoxicity to Raw264.7 cells (Figure S15). To assess whether low‐dose PEG‐Cu‐SAzyme is competent as an antioxidant to protect cells, we incubated Raw264.7 cells with tert‐butyl hydroperoxide (tBHP) to increase intracellular O_2_^•−^. As shown in Figure 4B, the pre‐incubated low‐dose PEG‐Cu‐SAzyme prevented the oxidative death of cells caused by tBHP in a concentration‐dependent manner. To further simulate the cell microenvironment of sepsis, we used an in vitro lipopolysaccharide (LPS)‐induced inflammation model. Compared with the LPS‐treated control group, the intracellular O_2_^•−^ level of the PEG‐Cu‐SAzyme‐treated group experienced a significant reduction, with an effect similar to the SOD‐treated group (Figure 4C). Moreover, the DCFH‐DA probe detected a large amount of ROS in Raw264.7 cells in the LPS treatment group (Figure 4D,E). In contrast, under the treatment of PEG‐Cu‐SAzyme, the DCF fluorescence intensity in the cells was significantly reduced, suggesting the effective antioxidant effect of PEG‐Cu‐SAzyme. PEG‐Cu‐SAzyme also significantly reduced the oxidative damage of DNA, as demonstrated by the detection of phosphorylated γ‐H_2_AX foci (Figure 4F,G). Finally, we evaluated the levels of pro‐inflammatory cytokines of tumor necrosis factor (TNF‐α) and interleukin 6 (IL‐6) secreted by inflammatory model cells. As shown in Figure 4H,I, LPS induction significantly increased the secretion levels of TNF‐α and IL‐6 in cells, but this process was prevented by pre‐treating with PEG‐Cu‐SAzyme. All the above results indicate that PEG‐Cu‐SAzyme, as a SOD‐like nanozyme, is effective in anti‐oxidative stress and anti‐inflammatory in the redox cycle of sepsis.

### 2.4 Treatment of sepsis in vivo with therapeutic Cu‐SAzyme

**FIGURE 5:**
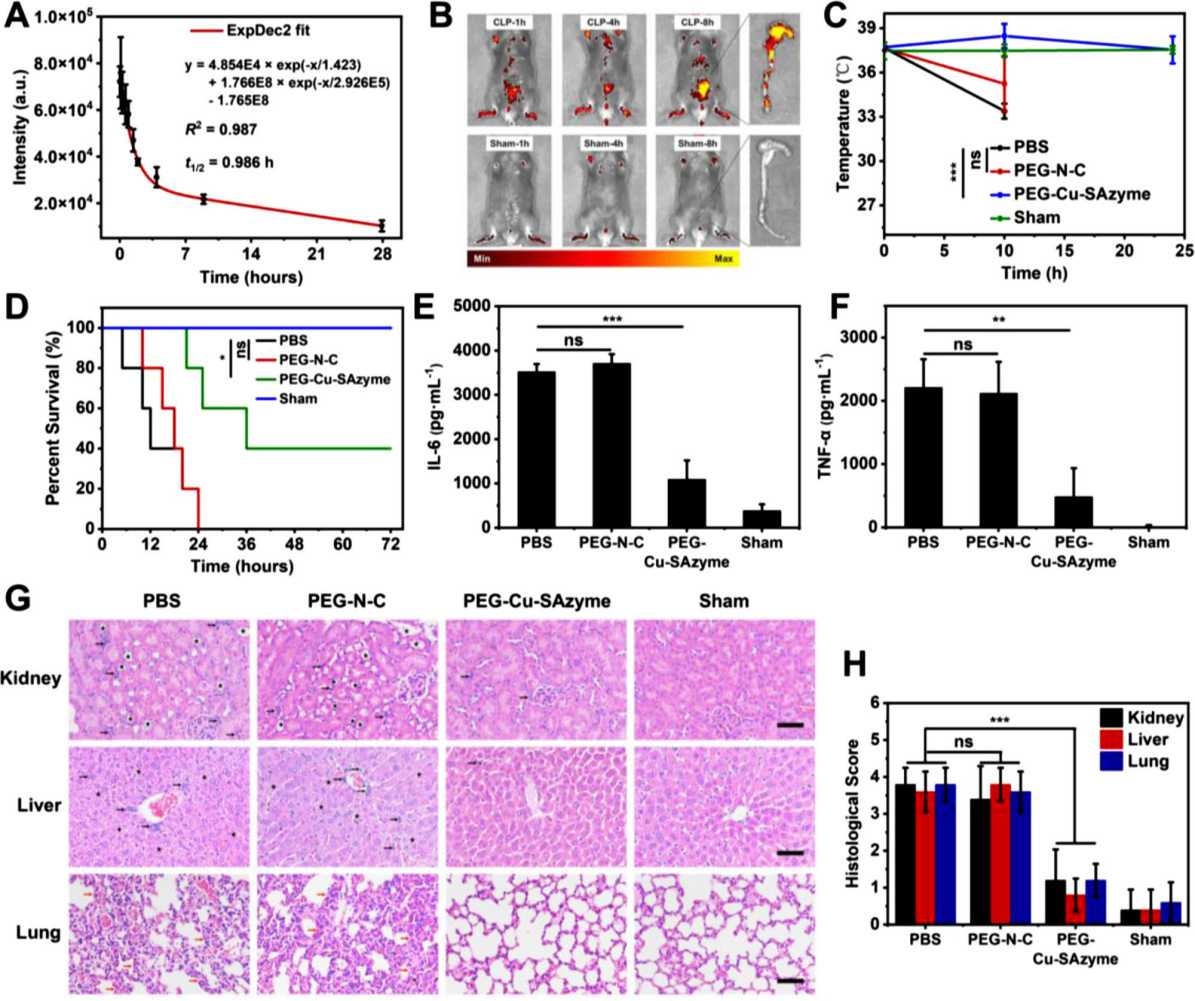
Antioxidant and anti‐inflammatory ability of PEG‐modified Cu‐SAzyme nanozymes in cells. (A) The blood circulation curve of intravenously injected PEG‐Cu‐SAzyme nanozymes. (B) In vivo near‐infrared fluorescence optical images of CLP models or Sham mice 1, 4, and 8 h after injection of Cy5.5‐labeled PEG‐Cu‐SAzyme nanozymes. The inset shows the fluorescence images of the cecum dissected 8 h after injection. Colored signals denote Cy5.5 fluorescence (Ex = 675 nm and Em = 720 nm). (C) The body temperature changes of CLP models in different treatment groups. (D) Survival curves of different groups of CLP models observed for 3 days. (E) The level of cytokine IL‐6 in the serum of CLP models in different groups. (F) The level of cytokine TNF‐α in the serum of CLP models in different groups. (G) Representative H&E staining images of the kidney, liver, and lung of septic mice in different treatment groups. Scale bar, 200 µm. Black arrows mark focal inflammatory cell infiltration, black pentagrams mark intracellular vacuolation, and red arrows indicate thickened alveolar septa. (H) Histological score of the obtained H&E staining images based on the severity of the injury (range 0–4)

To verify whether the sepsis treatment effect of Cu‐SAzyme consistently manifests in vivo, a cecal ligation and puncture (CLP) mouse model was constructed, which could reproduce the main features of complex clinical sepsis. We first analyzed the biocompatibility and pharmacokinetics of PEG‐Cu‐SAzyme. PEG‐Cu‐SAzyme showed a negligible rate of hemolysis (Figure S16), indicating that it is competent to be safely administered intravenously. The blood circulation half‐life of PEG‐Cu‐SAzyme was determined to be 0.986 h (Figure 5A), ensuring sufficient time for it to reach the lesion site and perform catalysis. Immediately after CLP induction or sham operation, Cy5.5‐labeled PEG‐Cu‐SAzyme was injected into mice via the tail vein and the near‐infrared fluorescence signal was measured serially. The fluorescence signal of Cy5.5‐PEG‐Cu‐SAzyme was detected to be enriched and distributed in the abdomen of CLP mice, especially near the cecum (Figure 5B). As a control, a little signal was detected in the abdomen and cecum of sham‐operated mice. Quantitative analysis of the region of interest (ROI) showed that the fluorescence signal of Cy5.5‐PEG‐Cu‐SAzyme gradually accumulated over time in the abdomen of CLP mice (Figure S17). These results suggest that PEG‐Cu‐SAzyme can easily be diffused or infiltrated into the damaged tissue and act locally at the lesion site in the early stage of infection, a phenomenon that has also been reported in several other studies.^[^ ^40^, ^62^ ^]^

The body temperature of CLP mice administered with PBS or N‐C dropped sharply (Figure 5C), indicating that the mice were experiencing severe CLP sepsis.^[^ ^63^ ^]^ The body temperature of CLP mice in the PEG‐Cu‐SAzyme‐treated group remained constant, demonstrating a moderate CLP symptom. Additionally, within 24 h of CLP induction, PEG‐Cu‐SAzyme‐administered mice maintained a survival rate of 80%, while all mice in the PBS or N‐C treatment group died (Figure 5D). After 3 days of treatment, the median survival rate of mice in the PEG‐Cu‐SAzyme treatment group still held at 40%, suggesting that PEG‐Cu‐SAzyme effectively reduced the mortality rate. Along with the improved survival rate, serum pro‐inflammatory cytokine concentrations such as TNF‐α and IL‐6 levels were also significantly reduced after PEG‐Cu‐SAzyme treatment compared with the control group (Figure 5E,F). In the further analysis by hematoxylin and eosin (H&E) staining, septic mice exhibited severe inflammatory cell infiltration in the kidneys, livers, and lungs, showing typical multi‐organ damage, such as vacuolation in kidney and liver, thickening of alveolar septa, etc., while reduced inflammatory response appeared in the PEG‐Cu‐SAzyme‐treated group (Figure 5G,H). These results indicate that SOD‐like Cu‐SAzyme is sufficient as an antioxidant and anti‐inflammatory agent for the treatment of sepsis.

## 3 CONCLUSION

In summary, we successfully de novo‐designed the SOD‐inspired single‐atom nanozyme (Cu‐SAzyme) with atomically dispersed Cu‐N_4_ active sites. The as‐synthesized Cu‐SAzyme exhibits an excellent SOD‐like activity, which effectively eliminated O_2_^•−^, the source of multiple RONS. As an antioxidant, Cu‐SAzyme effectively relieved the overall oxidative stress in activated inflammatory cells. Moreover, Cu‐SAzyme with excellent SOD activity alleviates the systemic inflammatory response and ROS‐induced multi‐organ dysfunction and therefore significantly prolongs the survival time of septic animals. Considering the current situation where there is no satisfactory medicine for fatal sepsis, Cu‐SAzyme holds the potential to be a promising antioxidant and anti‐inflammatory drug for the treatment of sepsis.

## 4 EXPERIMENTAL SECTION

Experimental details are provided in the Supporting Information.

## CONFLICT OF INTEREST

Kelong Fan and Xiyun Yan are members of the *Exploration* editorial board. All authors declare no conflict of interests.

## ETHICS STATEMENT

The animal experimental procedure was conducted with the approval of the Institutional Animal Care and Use Committee of the Institute of Biophysics, Chinese Academy of Sciences (approval number: SYXK2019021).

## Supporting information



Supporting Information

## Data Availability

The authors declare that all data needed to support the finding of this study are presented in the article and the Supporting Information. The data that support the findings of this study are available from the corresponding author upon reasonable request.
