# Supplementary material for: Bioinspired copper single‐atom nanozyme as a superoxide dismutase‐like antioxidant for sepsis treatment
Source: Exploration (Beijing). 2022 Jul 13;2(4):20210267. doi: 10.1002/EXP.20210267 (PMC10191017; doi:10.1002/EXP.20210267)
Supplement: Supplementary file 1 — Supporting Information [file EXP2-2-20210267-s001.docx]

Supporting Information

Bioinspired copper single-atom nanozyme as a superoxide dismutase-like antioxidant for sepsis treatment

Ji Yang,^1,3,4,‡^ Ruofei Zhang,^2,‡^ Hanqing Zhao,^2,5^ Haifeng Qi,^1,4^ Jingyun Li,^7^ Jian-Feng Li,^3^ Xinyao Zhou,^8^ Aiqin Wang,^1,4,*^ Kelong Fan,^2,5,6,*^ Xiyun Yan,^2,5,6,*^ Tao Zhang,^1,4^

1. ^1^ Collaborative Innovation Center of Chemistry for Energy Materials (iChEM), Dalian Institute of Chemical Physics, Chinese Academy of Sciences, Dalian 116023, China.
2. ^2^ CAS Engineering Laboratory for Nanozyme, Key Laboratory of Protein and Peptide Pharmaceutical, Institute of Biophysics, Chinese Academy of Sciences, Beijing 100101, China.
3. ^3^ Collaborative Innovation Center of Chemistry for Energy Materials (iChEM), College of Chemistry and Chemical Engineering, Xiamen University, Xiamen 361005, China.
4. ^4^ CAS Key Laboratory of Science and Technology on Applied Catalysis, Dalian Institute of Chemical Physics, Chinese Academy of Sciences, Dalian, 116023, China.
5. ^5^ University of Chinese Academy of Sciences, Chinese Academy of Sciences, Beijing 100408, China.
6. ^6^ Nanozyme Medical Center, School of Basic Medical Sciences, Zhengzhou University, Zhengzhou 450052, China.
7. ^7^ Key Laboratory of Infection and Immunity, Institute of Biophysics, Chinese Academy of Sciences, Beijing 100101, China.
8. ^8^ School of Engineering and Applied Science, University of Pennsylvania, Philadelphia, 19104, USA.
9. ^‡^Ji Yang and Ruofei Zhang contributed equally to this work.

**Materials and methods**

**Materials.** 1,2-Distearoyl-sn-glycero-3-phosphoethanolamine-polyethylene glycol (2000)-amine (DSPE-PEG (2000)-NH_2_) was purchased from ToYang Bio (Shanghai, China). Fluorescein isothiocyanate (FITC) isomer I, dimethyl sulfoxide (DMSO), xanthine, xanthine oxidase, penicillin, streptomycin, superoxide anion assay kit, lipopolysaccharide (LPS), phorbol-12-myristate-13-acetate (PMA), 2',7'-dichlorofluorescein diacetate (H2DCFDA, Catalog#: D6883), paraformaldehyde (PFA), and Triton X-100 were purchased from Sigma-Aldrich Inc. (St Louis, Missouri, USA). Cy5.5 mono NHS ester was purchased from Amersham Biosciences (Buckinghamshire, UK). Fetal bovine serum (FBS) was purchased from Tianhang Biotechnology Co.,Ltd. (Hangzhou, Zhejiang, China). 4',6'-dimidyl-2-phenyl (DAPI) was purchased from Roche Applied Science (Indianapolis, IN, USA). Rabbit anti-γ-H2AX monoclonal antibody was purchased from Bethyl Laboratories (Catalog#: A700-053, Montgomery, TX). Cy5 labeled goat anti-rabbit secondary antibody was purchased from Invitrogen (Catalog#: A10523, Thermo Fisher Scientific, USA). Mouse TNF-α ELISA and IL-6 enzyme-linked immune sorbent assay (ELISA) kits were purchased from Boster Biotechnology (Wuhan, China).

**Synthesis of N-doped C.** 2,6-Diaminopyridine (2,6-DAP, 5.45 g; 98%, Aladdin), 40wt% LU-DOX colloidal silica (SiO_2_, 3 mL; Sigma-Aldrich) were dispersed in ultrapure water (400 mL; 18.2 MΩ cm) under sonication for 30 min to form a homogenous solution. After that, NaOH (1 g; TianDa Chemical Reagent) was dissolved into the above solution for another 10 min stirring. Subsequently, the ammonium persulfate solution (APS, 17.13 g dissolved in 100 mL H_2_O; Aladdin) served as initiator was added into the dispersion immediately with vigorous stirring for 8min, and then followed by a slow stirring for 12 h. Noting that the all process above was conducted in ice bath. The as-prepared products of polymeric diaminopyridine (PDAP) were collected by filtered and dried overnight in oven at 80 °C to get a brown solid which was denoted as SiO_2_@PDAP. The resulting polymers was further annealed under flowing He/NH_3_ mixture (V : V= 2:1, 75 mL/min) for 2 h at 800 °C with a heating rate of 5 °C/min to obtain a black carbonized product marked as SiO_2_@N-doped C. Afterwards, the obtained composite underwent 4% HF (Caution: danger!!!) etching to remove SiO_2_ template for 12 h, and then washed repeatedly with water. Finally, the etched sample was dried at 80 °C overnight to form N-doped C support.

**Synthesis of Cu-SAzyme.** Typically, a certain mass of Cu(NO_3_)_2_·3H_2_O was firstly dissolved in ethanol (50 mL) at 60 °C. Then, the as-prepared N-doped C (100 mg) was dispersed in the solution above and then sonicated for 30 min to form a homogeneous mixture. Subsequently, the resulting dispersion was kept at 60 °C under continuous magnetic stirring for 12 h. Finally, this catalyst was collected by filtered with ethanol and H_2_O, and then dried overnight at 80 °C.

**Synthesis of Cu NPs/N-C.** 5.45 g monomer of 2,6-Diaminopyridine and 1.2 g SiO_2_ powder were dispersed in 200 mL ultrapure water for 30 min under sonication, another 50 mL aqueous solution containing 6 g Cu(NO_3_)_2_·3H_2_O was added into above at one time. After stirring for 12 h, the brown solid products were collected by rotating evaporation and dried overnight at 60 °C. Afterwards, the obtained products were subjected to calcination at 800 °C under N_2_ flow for 2 h, and subsequently, the resulted black powders were subjected to acid treatment to remove silica and exposed metal aggregation.

**Physical characterizations.** X-ray diffraction (XRD) pattern for Cu-SAzyme was recorded on a PANalytical X’pert diffractometer using nickel-filtered Cu Kα radiation to characterize the structure. The metal content of all catalysts was quantitated by inductively coupled plasma optical emission spectroscopy (ICP-OES) (7300 DV, PerkinElmer). Noting that Cu-N-C samples was firstly annealed under air in muffle furnace at 600 °C for 2 h to obtain metal oxides, and then underwent acid treatment (Caution: concentrated HCl + HNO_3_ mixture!!!) to get a homogeneous solution before ICP analysis. The surface configuration of catalysts was analysed by X-ray photoelectron spectroscopy (XPS) on a Thermo ESCALAB 250 Xi spectrometer equipped with an Al anode (Al Kα= 1846.6 eV), operated at 10.8 mA and 15 kV. Raman data was acquired on Xplora Plus Raman instrument using He-Ne laser at 632.8 nm. And the intensity ratio of D and G peak (I_D_/I_G_) was calculated to unveil the graphitization degree of catalysts. The obtained nitrogen adsorption-desorption isotherms carried out on a Micromeritics ASAP 2460 apparatus (Quadrasorb SI Automate Surface Area & Pore Size Analyzer) at -196 °C was analysed to evaluate the specific surface areas. The scanning electron microscopy (SEM) was employed to characterize the structural morphologies of catalysts, operated at 15.0 kV on a field-emission JSM-7800F. The high-resolution TEM, STEM images and elemental mapping were performed in a JEOL-2100F microscope at 200 kV. Aberration-corrected high-angle annular dark field scanning transmission electron microscopy (AC-HAADF-STEM) images were taken on JEOL JEM-ARM200F STEM/TEM system with an atomic resolution of 0.08 nm. The both XANES and EXAFS data of Cu K-edge were collected under ambient conditions in transmission mode at the 14W1 beam line of Shanghai Synchrotron Radiation Facility (SSRF), using a Si(111) double-crystal monochromator. The XAFS data was further analysed by Athena software which the energy was calibrated using Cu foil.

**Synthesis of polyethylene glycol (PEG) modified Cu-SAzyme.** To synthesize PEG-Cu-SAzyme, 5 mL of Cu-SAzyme sample and DSPE-PEG (2000)-NH_2_ were sonicated for 2 h in an ice bath. After that, the suspension was centrifuged at 12,000 rpm for 10 min, and the precipitate was washed three times with deionized water to remove excess DSPE-PEG (2000)-NH_2_. The synthesized PEG-Cu-SAzyme sample was finally dispersed in deionized water.

**Synthesis of FITC or Cy5.5-conjugated PEG-Cu-SAzyme.** To obtain FITC or Cy5.5-NHS ester-conjugated PEG-Cu-SAzyme, 5 mg of the selected dye was first dissolved in 100 μL DMSO, and then mixed with PEG-Cu-SAzyme in a carbonate buffer (pH 9.0) at 4 °C overnight. In this process, FITC or Cy5.5-NHS ester covalently bond with the amino groups on the surface of PEG-Cu-SAzyme. After that, the suspension was centrifuged at 12,000 rpm for 10 min, and the precipitate was washed with deionized water 3 times to remove unbound free dye. The finally obtained fluorescently labeled PEG-Cu-SAzyme was dispersed in deionized water.

**SOD-like activity of Cu-SAzyme.** The SOD-like activity of Cu-SAzyme was evaluated by a SOD assay kit (Dojindo Laboratories, Japan). First, 20 µL Cu-SAzyme solutions with different concentrations were mixed with 200 µL of WST-1 working solution. The reaction was initiated after the addition of 20 µL of xanthine oxidase solution. After mixing at 37 °C for 20 min, the absorbance at 450 nm was detected using a Molecular Devices M5 Multimode Microplate Reader (USA). Under these specified conditions, the amount of Cu-SANzyme required to inhibit a 50% reduction in formazan formation was defined as 1 unit of SOD-like activity.

The inhibition rate was calculated according to the following formula:

Inhibition rate (%)= ((A_blank1_ - A_blank3_) - (A_sample_ - A_blank2_))/(A_blank1_ - A_blank3_) × 100%

Wherein, blank 1 is a blank control group without nanozyme samples, blank 2 is a blank control group without xanthine oxidase, and blank 3 is a blank control group without both nanozyme samples and xanthine oxidase.

The inhibition rates corresponding to various concentrations of nanozyme samples were measured respectively, and the half-inhibitory concentration (IC50) was calculated using the Dose-Response-Inhibition functional analysis in the GraphPad Prism 7 software.

Afterwards, the SOD specific activity of the nanozyme was calculated using the following formula:

SOD activity (U/mg)= 1 (U) / (IC50(mg/mL) * V(mL))

Wherein, V is the volume of nanozyme used.

**Cell culture.** The Murine macrophage cell line Raw 264.7 and the Human leukemic monocyte lymphoma cell line U937 cells were purchased from American Type Culture Collection (ATCC). Raw 264.7 cells were cultured in high glucose Dulbecco’s modified Eagle’s medium (DMEM) at 37 °C in a humidified 5% CO_2_ atmosphere. U937 cells were cultured in Roswell Park Memorial Institute (RPMI) 1640 medium. All cell growth media were supplemented with 10% heat-inactivated FBS, penicillin (100 U/mL) and streptomycin (100 μg/mL).

***In vitro* endocytosis assay.**

To study endocytosis, Raw 264.7 cells were seeded on a confocal culture dish (coverslip bottom dish) at a concentration of 5×10^4^ cells/well and cultured for 24 h. Afterwards, the adherent cells were treated with FITC-conjugated PEG-Cu-SAzyme (10 μg/mL) treatment. After 1 h, 2 h, and 4 h, the cells were washed with PBS three times to remove free particles. Fluorescence signals were collected with a Zeiss LSM700 laser confocal fluorescence microscope. Quantitative analysis of FITC intensity was performed using ImageJ Analysis Software (National Institutes of Health, Bethesda, MD).

***In vitro* superoxide anion assay.** A superoxide anion assay kit was used to evaluate the superoxide anion scavenging ability of Cu-SAzyme. The assay was carried out according to the instructions of the kit. Briefly, U937 cells were cultured for 24 h and then stimulated by LPS (1 μg/mL) for 48 h. The treated cells were divided equally into a 96-well plate with 1×10^6^ cells per well. The reaction components mixed with PMA, enhancer solution, lumino solution and assay buffer were added to each well to initiate the reaction. At the same time, various concentrations of Cu-SAzyme were added to the treatment group. The 96-well plate was shaken to mix the reaction components, and the luminescence was detected with a microplate reader every 5 min for 4 h.

**Intracellular ROS scavenging by Cu-SAzyme**. The intracellular ROS level was assessed using a fluorescent probe H2DCFDA, which is non-fluorescent but could react with intracellular ROS and generate the fluorescent product of dichloro-fluorescein (DCF). To evaluate the ability of Cu-SAzyme to eliminate intracellular ROS, Raw 264.7 cells were seeded on a confocal culture dish (coverslip bottom dish) at a concentration of 5×10^4^ cells/well and cultured for 24 h. The cell medium was removed, and then adherent cells were stimulated with 2 μg/mL LPS for 12 h with or without co-treatment of PEG-Cu-SAzyme (10 μg/mL) in high glucose DMEM with 10% FBS at 37 °C. After that, the adherent cells were washed three times with PBS to remove excess LPS and PEG-Cu-SAzyme. Then, H2DCFDA solution (10 μM in phenol red free DMEM) was added to the cells and incubated at 37 °C for 30 min. The nuclei of cells were stained with DAPI (1 μg/mL) at 37 °C for 10 min. After washing away the free probe with PBS, the fluorescence intensity of the cells was monitored using a laser confocal fluorescence microscope. Quantitative analysis of DCF intensity was performed using ImageJ Analysis Software (National Institutes of Health, Bethesda, MD).

**Assessment of DNA damage.** To analyze DNA damage, Raw 264.7 cells were seeded on a confocal culture dish (coverslip bottom dish) at a concentration of 5×10^4^ cells/well and cultured for 24 h. The cell medium was removed, and then adherent cells were stimulated with 2 μg/mL LPS for 12 h with or without co-treatment of PEG-Cu-SAzyme (10 μg/mL) in high glucose DMEM with 10% FBS at 37 ^o^C. After that, immunofluorescence staining was performed to analyze the γ-H_2_AX foci. Firstly, the cells were washed with PBS, fixed with 4% PFA for 30 min, and then permeabilized with 0.5% Triton X-100 for 10 min. Afterwards, the cells were incubated with 5% goat serum diluted in PBS at 37 °C for 30 min to block other non-specific protein interactions. After that, the cells were incubated with rabbit anti-mouse γ-H2AX monoclonal antibody (1:200) at 37 °C for 2 h. After washing three times with PBS, the cells were subsequently incubated with Cy5 labeled goat anti-rabbit secondary antibody at 37 °C in the dark for 45 min. The unbound fluorescent secondary antibody was washed away with PBS. The nuclei were stained with DAPI for 10 min at room temperature. Finally, the fluorescence intensity of the cells was analyzed using a laser confocal fluorescence microscope. Quantitative analysis of γ-H_2_AX foci was performed using ImageJ Analysis Software (National Institutes of Health, Bethesda, MD).

***In Vitro* Cytokine Assays.** Raw 264.7 cells cultured in 6-well plates were stimulated with LPS (1 μg/mL) for 24 h with or without the cotreatment of PEG-Cu-SAzyme (10 μg/mL) in high glucose DMEM with 10% FBS at 37 °C and 5% CO_2_. After that, the cell culture supernatant was collected and centrifuged at 12000 rpm at 4 °C for 15 min. The concentration of TNF-α and IL-6 was determined using ELISA kits following instructions recommended by the manufacturer. Briefly, the test samples and standard samples were added to the ELISA plate pre-coated with IL-6 or TNF-α monoclonal antibody, and reacted at 37 °C for 90 min. After that, biotin-labeled polyclonal antibodies were added and reacted at 37 °C for 60 min. The unbound antibodies were washed away with washing buffer. Then, the avidin-peroxidase complex was added to the plate and reacted at 37 °C for 30 min. Finally, TMB solution was added to the plate for 15 min at 37 °C, and then the color reaction was terminated with the termination solution. Finally, the absorbance at 450 nm was detected by a microplate reader. The content of IL-6 and TNF-α in the sample was calculated with reference to the standard curve.

**Hemolysis test of PEG-Cu-SAzyme.** 1.5 mL whole blood was collected from the eyes of a C57BL/6 mouse with a tube containing 200 μL of citric acid anticoagulant. The collected blood was mixed with an appropriate amount of 0.9% saline and centrifuged at 2500 rpm for 5 min. After that, the supernatant was removed, and 2 mL of 0.9% saline was added to resuspend the blood cells, and the suspension was centrifuged at 2500 rpm for 5 min. After that, the supernatant was removed, and 2 mL of 0.9% saline was added to resuspend the blood cells, and the suspension was centrifuged at 2500 rpm for 5 min. This operation was repeated until the supernatant changed from opaque to colorless and transparent. The collected erythrocytes were resuspended in 1 mL of 0.9% saline and divided into 10 aliquots. Add 900 μL of distilled water or PEG-Cu-SAzyme (final concentration of 0-400 μg/mL) diluted with 0.9% saline to each aliquot. The erythrocytes suspension was dispersed by gently pipetting, and then incubated on a mixer for 4 h at room temperature. After that, the sample was centrifuged at 13000 rpm for 5 min, and the hemoglobin level in the supernatant was measured by analyzing the specific spectrophotometric absorption at 541 nm. The hemolysis rate (HR%) was calculated by the following formula.

HR%= (A_PEG-Cu-SAzyme_ - A_NC_) / (A_PC_ - A_NC_) × 100%

Where A_PEG-Cu-SAzyme_, A_PC_ and A_NC_ are the absorbance of the sample, the distilled water (positive control) and the 0.9% saline (negative control), respectively.

***In vivo* mouse model of sepsis induced by cecal ligation and puncture (CLP).** All animal studies were approved by the Institutional Animal Care and Use Committee of the Institute of Biophysics, Chinese Academy of Sciences (approval number: SYXK2019021). The CLP-induced bacteremia model was performed using 8-week-old C57BL/6 mice as described previously.^[1]^ In brief, after inhalational anesthesia with 3% isoflurane, the mice were made a 1 cm mid-abdominal incision and exposed the cecum. After that, the cecum was ligated with 5-0 polylactic acid (PLA) suture under the ileocecal valve, and the ligated cecum was punctured twice with a 22-gauge needle to cause fecal leakage inside the peritoneum, and the abdomen was closed. In the negative control group, a sham operation was performed under the same procedure but without ligation and puncture of the cecum.

***In vivo* near-infrared fluorescence optical imaging of Cy5.5-conjugated Cu-SAzyme.** Immediately after CLP-induced bacteremia or sham operation, Cy5.5-conjugated Cu-SAzyme was injected intravenously into mice via tail veins. *In vivo* near-infrared fluorescence imaging was performed with an IVIS Spectrum Imaging System (Caliper Life Sciences, Inc., Hopkinton, MA, USA) after various post-injection intervals (1, 4, and 8 h; excitation filter: 620 nm; emission filter: 710 nm). At each detection time point, the mice were anesthetized with isoflurane gas and laid down on their backs to collect fluorescence signals.

***In vivo* PEG-Cu-SAzyme treatment of sepsis.** To evaluate the therapeutic effect of PEG-Cu-SAzyme on sepsis, CLP-induced mice were divided into four groups: the PBS-treated group, the N-C-treated group, the Cu-SAzyme-treated group and the Sham group. Immediately after CLP induction, 200 μL of PBS, PEG-N-C (10 mg/kg) or PEG-Cu-SAzyme (10 mg/kg) were injected intravenously into the mice, while the mice in the Sham group were not treated with additional treatment. After the injection, the animals were monitored in real time for two days of body temperature and three days of survival.

**Histopathology investigation.** After 24 h of treatment, the main organs (liver, lung, kidney) of the mice in each group were collected and fixed with 4% PFA. The fixed organs were dehydrated by gradient ethanol and embedded in paraffin. The embedded tissue was sliced into 4μm-thick sections and stained with H&E. The histopathology was then analyzed under an optical microscope in a blinded fashion. Morphological changes in liver tissue were scored based on the Suzuki histological scoring system, according to the intensity of sinusoidal congestion, vacuolation of the hepatic cytoplasm, and parenchymal necrosis (0-no change; 1-minimal change, 2-mild change, 3-moderate change, 4-severe change).^[2]^ Renal injury was assessed using a renal injury scoring system based on the intensity of cytoplasmic vacuolation, cellular necrosis, tubular luminal debris, and obstruction (0-no change; 1-minimal change, 2-mild change, 3-moderate change, 4-severe change).^[3]^ Lung injury was scored based on morphological changes such as neutrophil infiltration, alveolar hemorrhage, and thickened alveolar septa (0-no change; 1-minimal change, 2-mild change, 3-moderate change, 4-severe change).^[4]^

***In vivo* detection of cytokine.** After 24 h of treatment, blood of mice in each group was collected and the serum was separated by centrifugation at 7000 g at 4 °C for 10 min. The levels of IL-6 and TNF-α cytokines in the serum were detected using an ELISA kit according to the manufacturer's instructions.

**Statistical analysis.** Data are shown as mean ± standard deviation (SD). Statistical significance was evaluated by an unpaired Student’s two-sided t-test: **P < 0.01, ***P < 0.001 and ****P < 0.0001.

**Supplementary Figures**

**
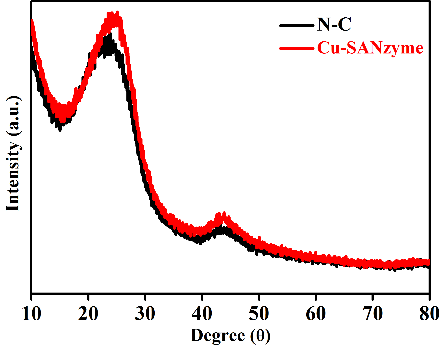
**

**Supplementary Figure 1.** The comparisons of XRD patterns between N-C and Cu-SAzyme.


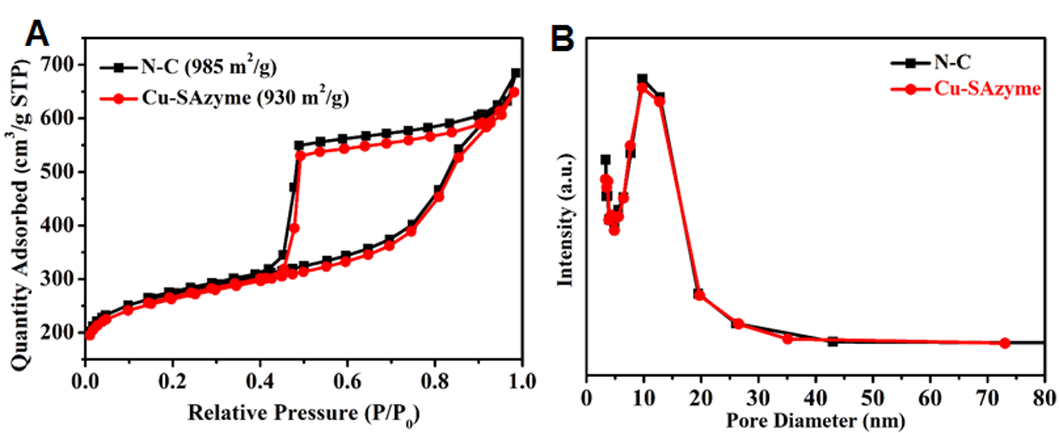


**Supplementary Figure 2.** The N_2_ adsorption-desorption isotherms (A), and the pore size distribution (B) of N-C and Cu-SAzyme.


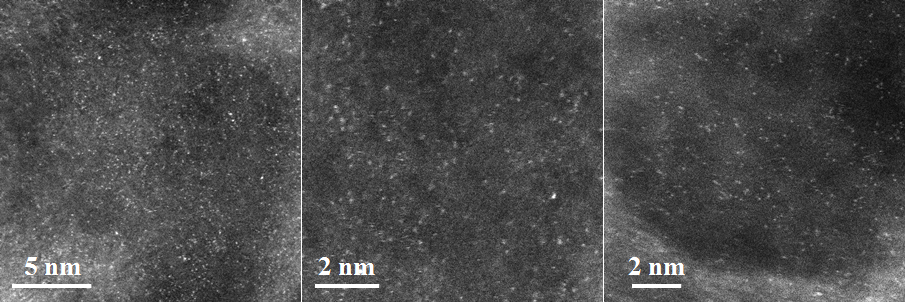


**Supplementary Figure 3.** HAADF-STEM images of Cu-SAzyme at different areas.


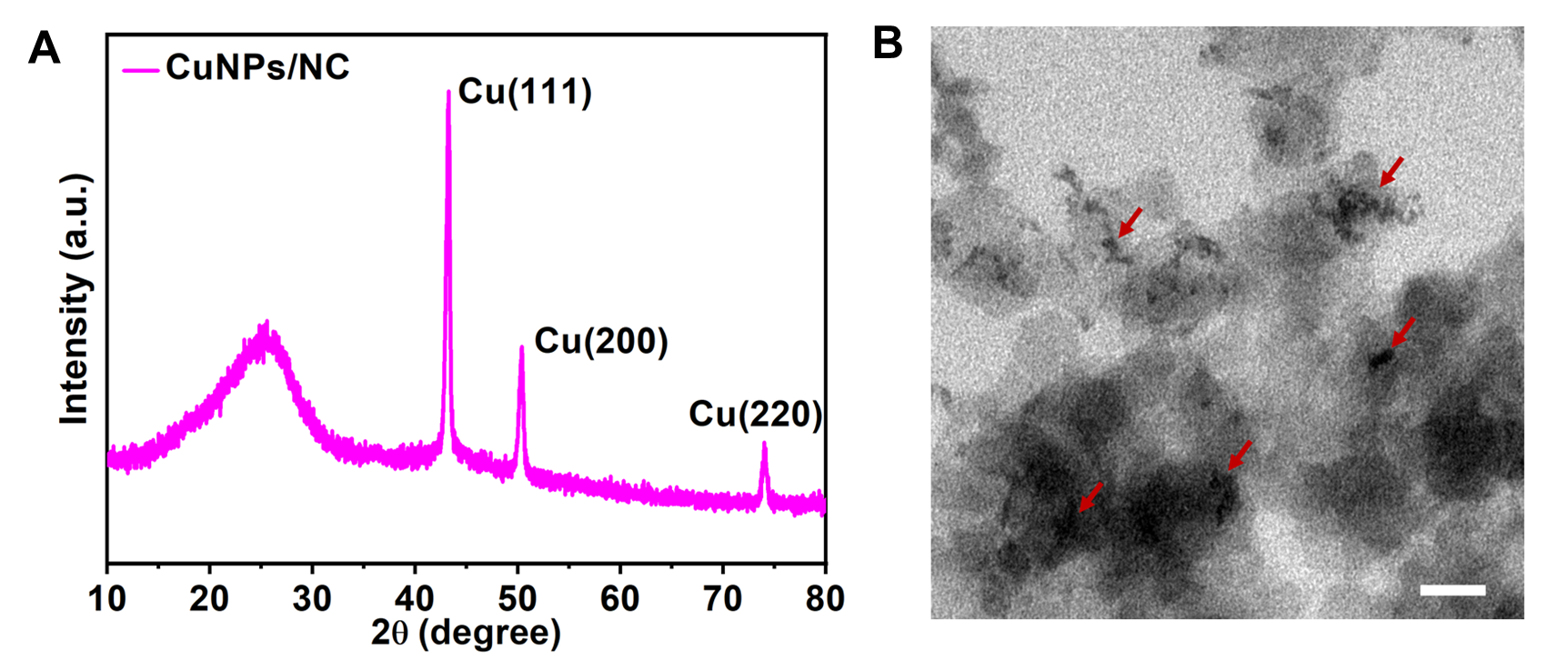


**Supplementary Figure 4.** The XRD pattern (A) and TEM image (B) of the one-pot synthetic sample (CuNPs/NC). Red arrows indicate metal aggregation. Scale bar, 50 nm.


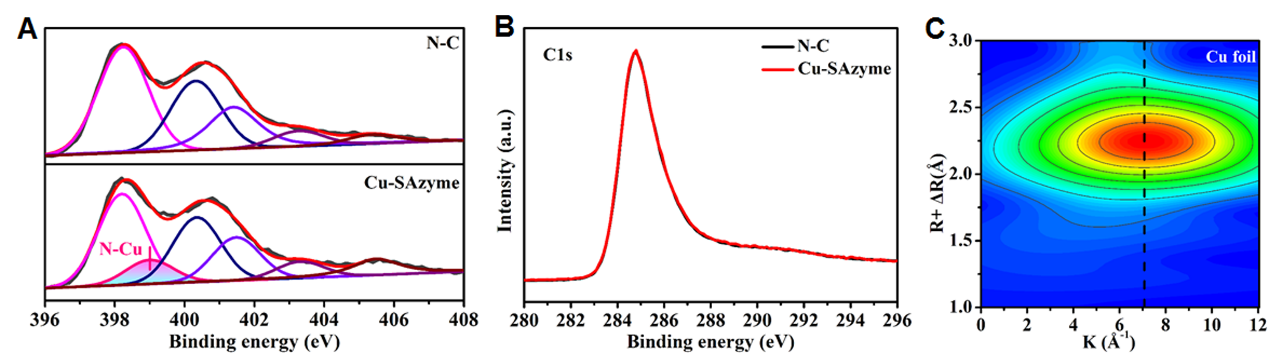


**Supplementary Figure 5.** A, high-resolution N1s spectra of N-C and Cu-SAzyme. B, high-resolution C1s spectra of N-C and Cu-SAzyme. C, WT plot of Cu foil.

**
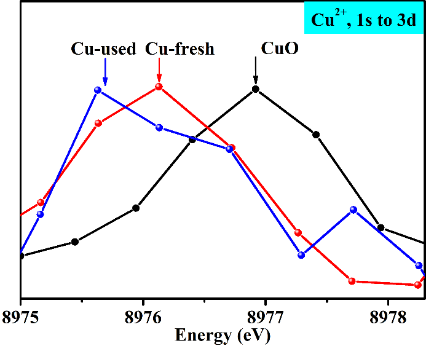
**

**Supplementary Figure 6.** The first derivative of 1s →3d Cu^2+^ peak of CuO and Cu-SAzyme.

**
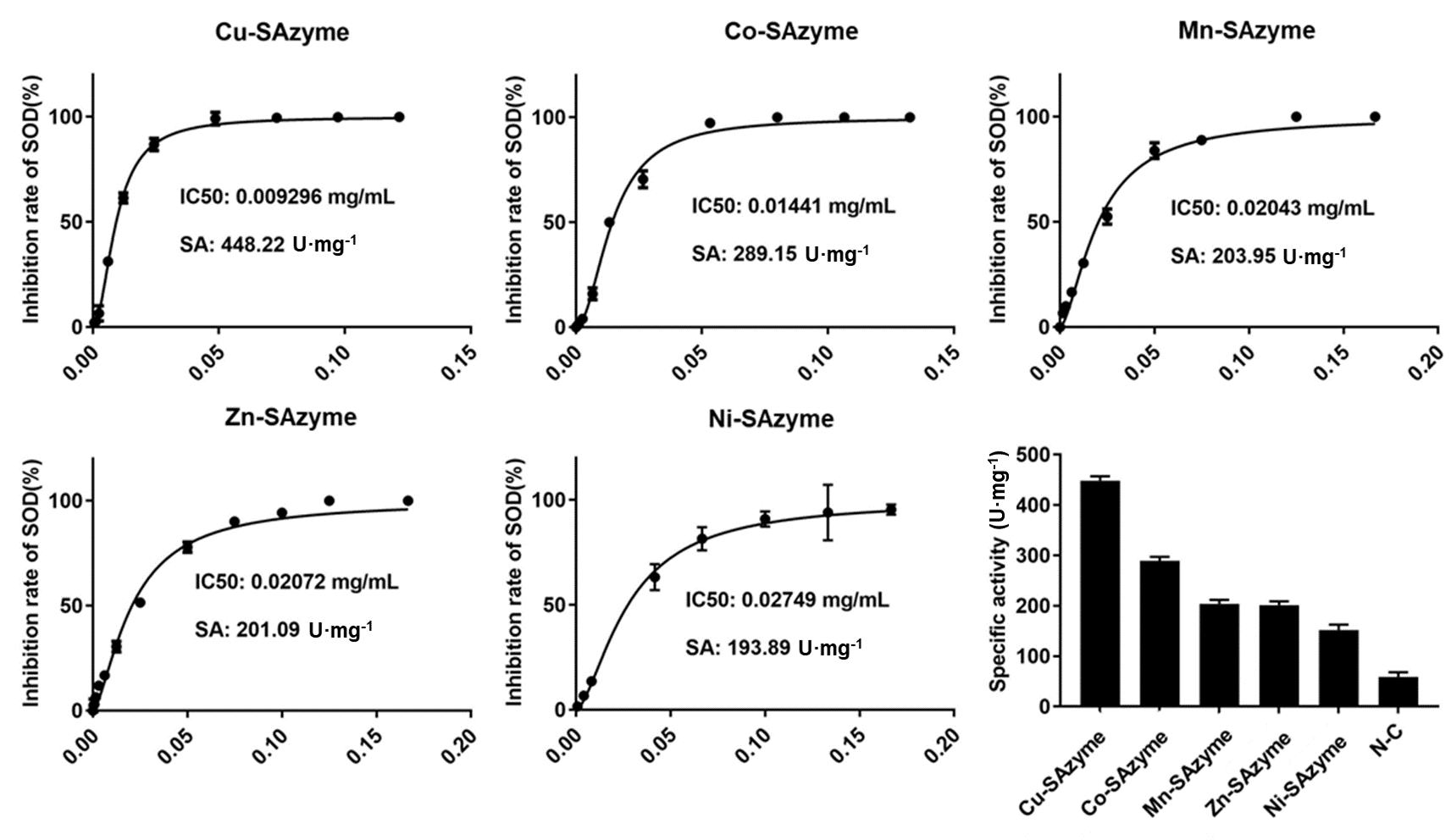
**

**Supplementary Figure 7.** SOD-like activities of different SAzymes synthesized with different metals.


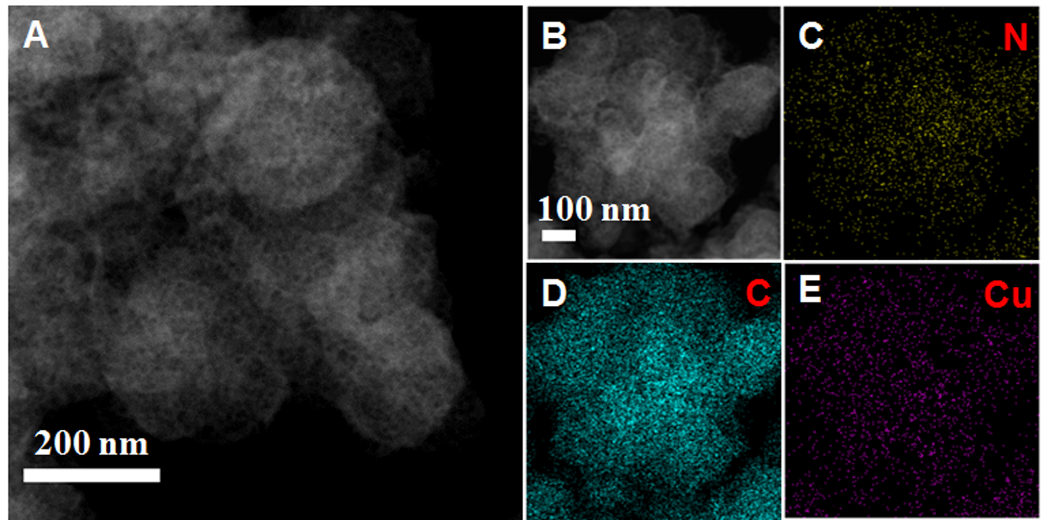


**Supplementary Figure 8.** STEM images and corresponding EDX-elemental mapping of the spent Cu-SAzyme.


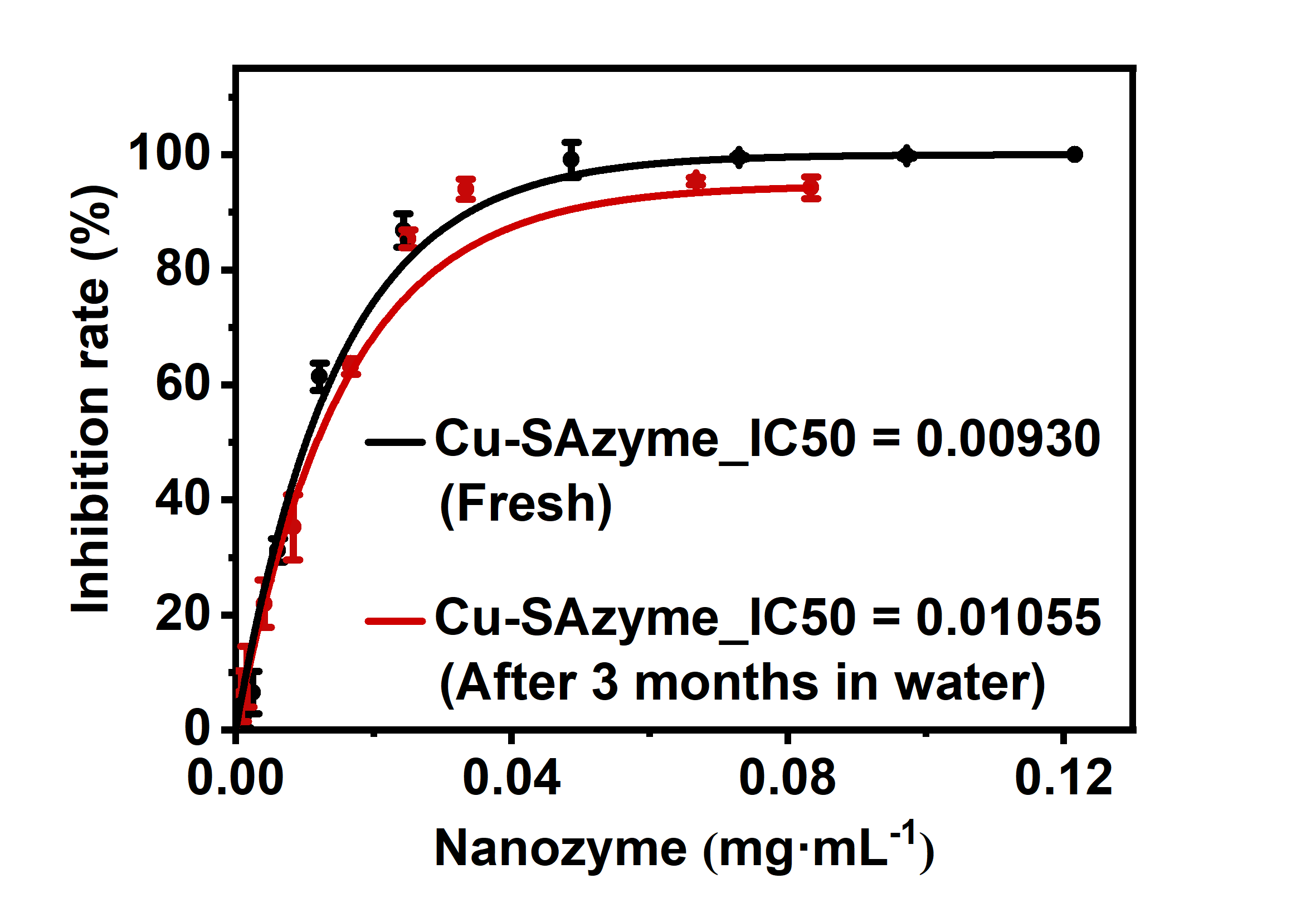


**Supplementary Figure 9.** Inhibition rate curve of fresh Cu-SAzyme (IC50= 0.00930 ± 0.00018, 448.22 ± 8.59 U·mg^-1^) and Cu-SAzyme after 3 months in water (IC50= 0.01055 ± 0.00048, 394.94 ± 17.18 U·mg^-1^).


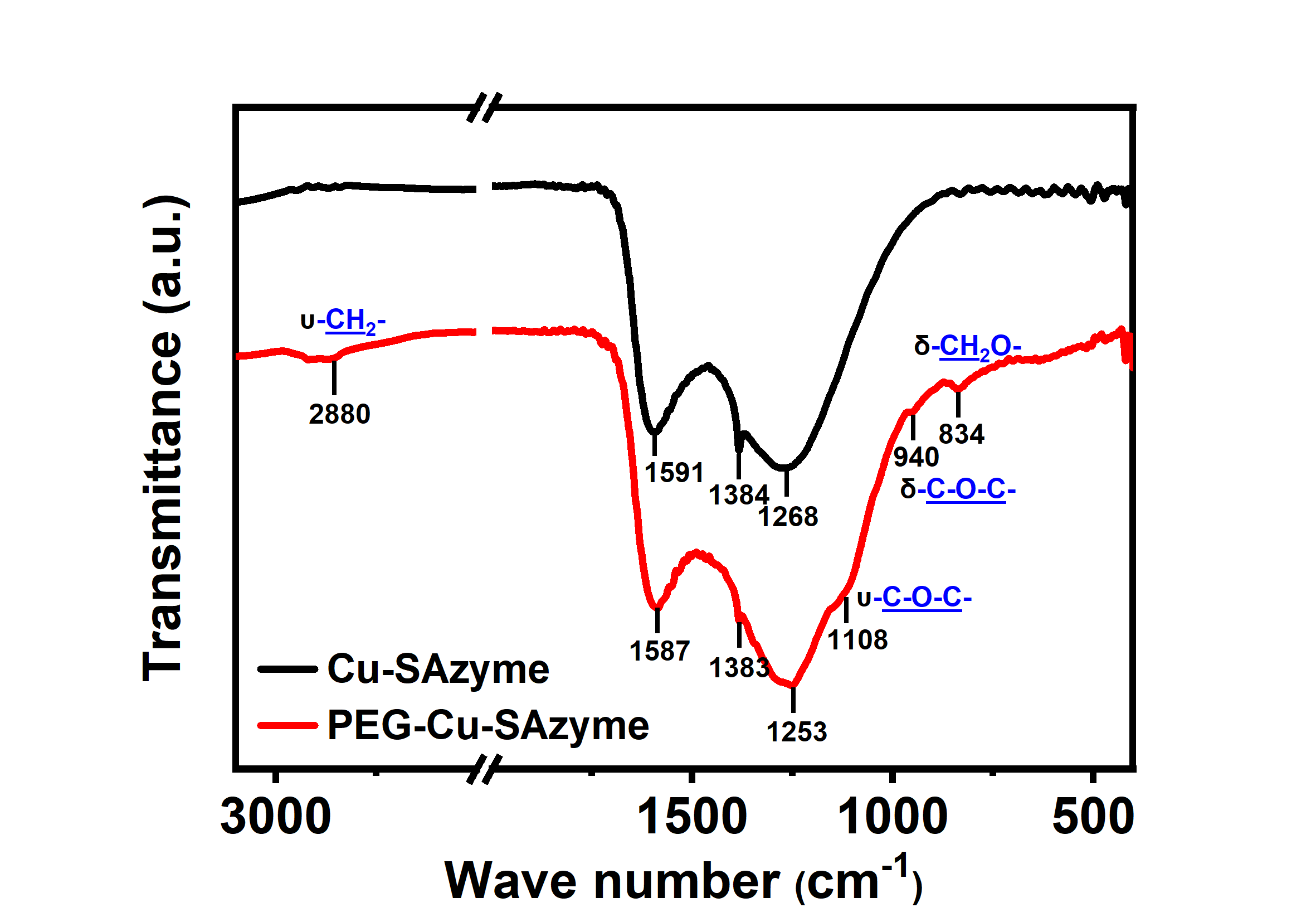


**Supplementary Figure 10.** FTIR spectra of Cu-SAzyme before and after PEG modification.


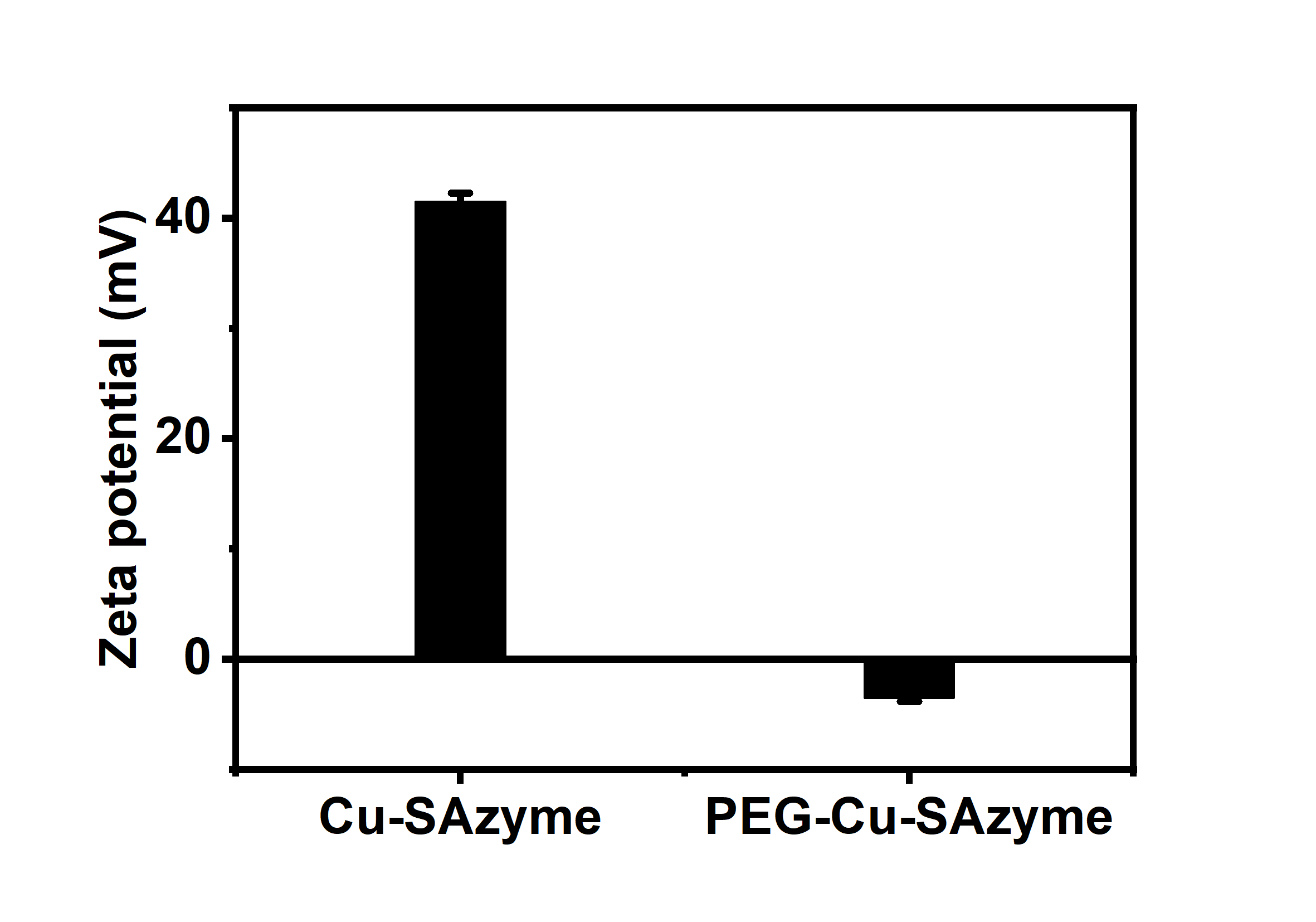


**Supplementary Figure 11.** The Zeta potential of Cu-SAzyme in water before and after PEG modification.


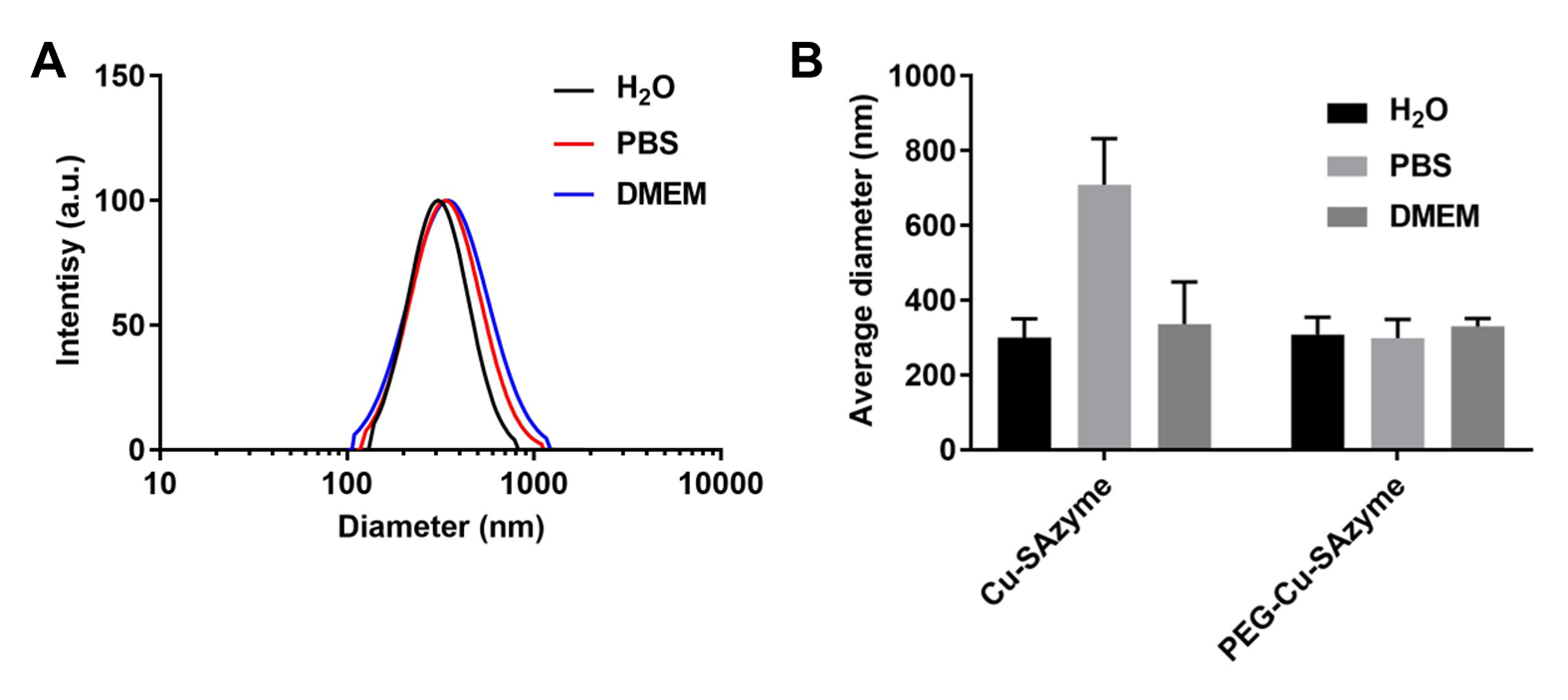


**Supplementary Figure 12**. DLS analysis of PEG-Cu-SAzyme. (A) Hydrodynamic diameter distribution of the PEG-Cu-SAzyme. (B) the average size of Cu-SAzyme and PEG-Cu-SAzyme in H_2_O, PBS or DMEM medium.


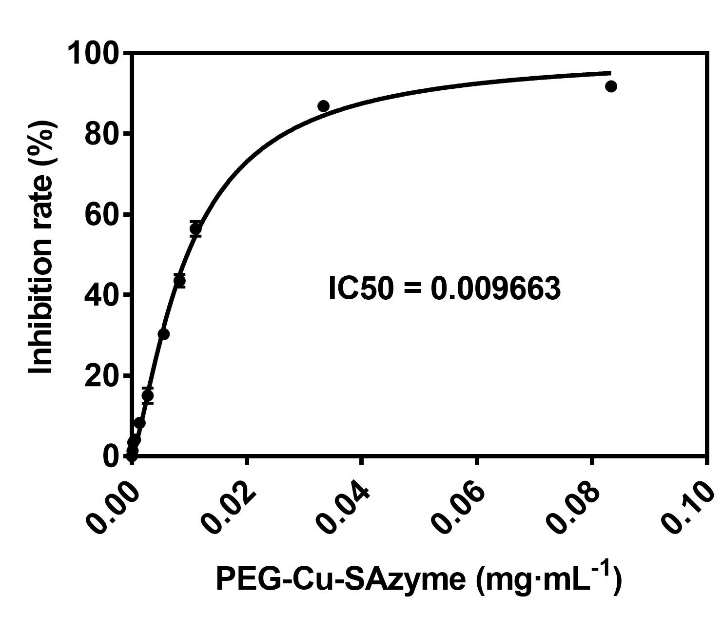


**Supplementary Figure 13.** Inhibition rate curve of PEG-Cu-SAzyme (IC50= 0.009668 ± 0.00020, 430.98 ± 8.74 U·mg^-1^).


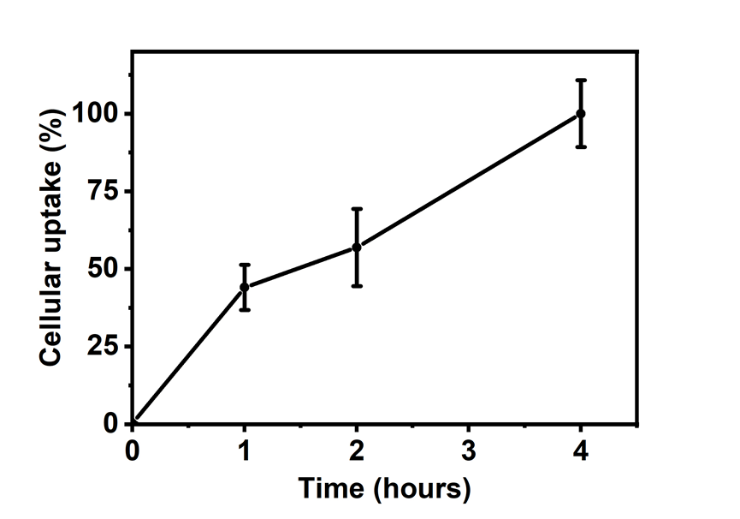


**Supplementary Figure 14.** Quantitative analysis of FITC-labeled PEG-Cu-SAzyme uptake by Raw264.7 cells within 0-4 hours.


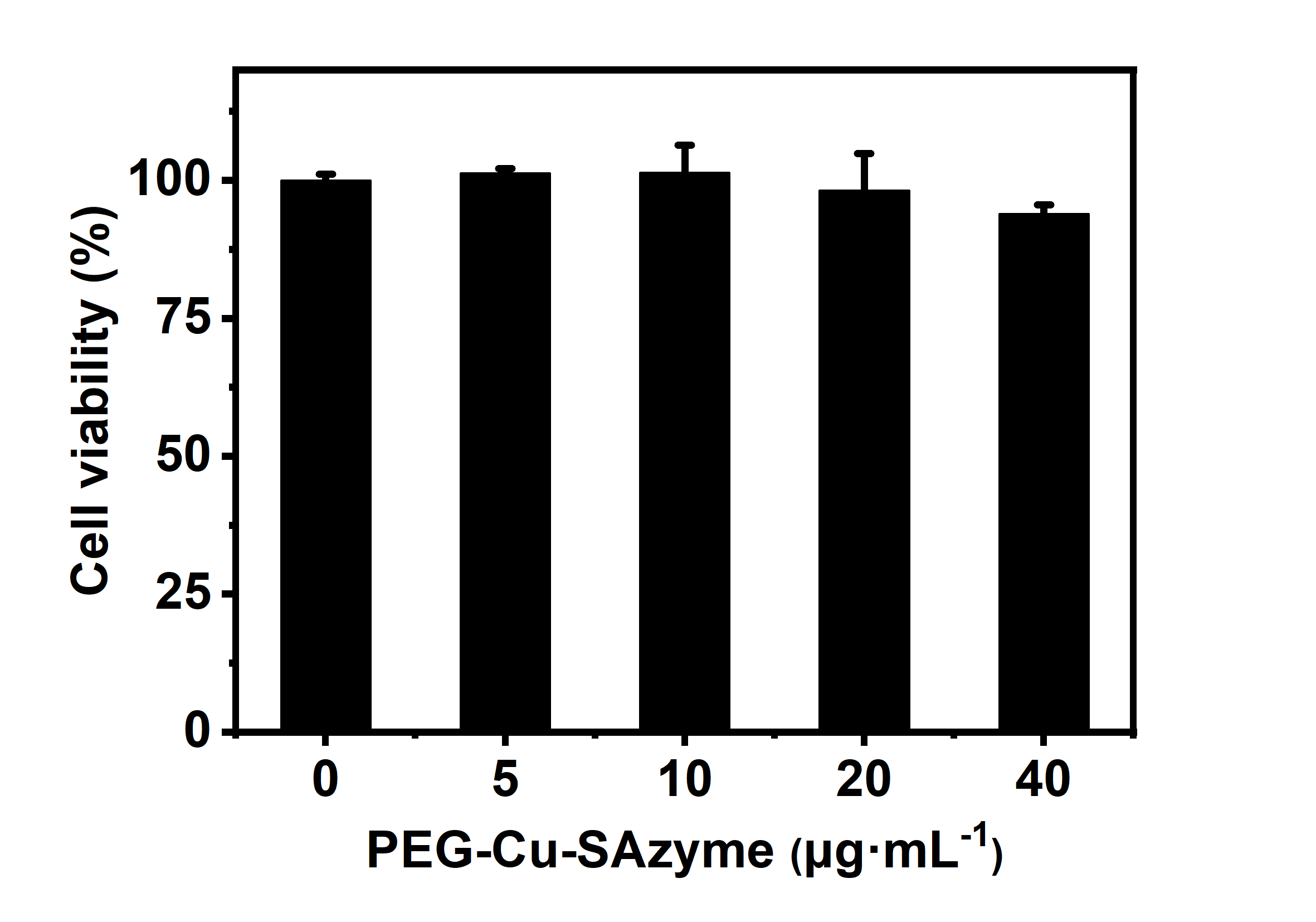


**Supplementary Figure 15.** The cell viability of Raw264.7 cells incubated with different concentrations of PEG-Cu-SAzyme.


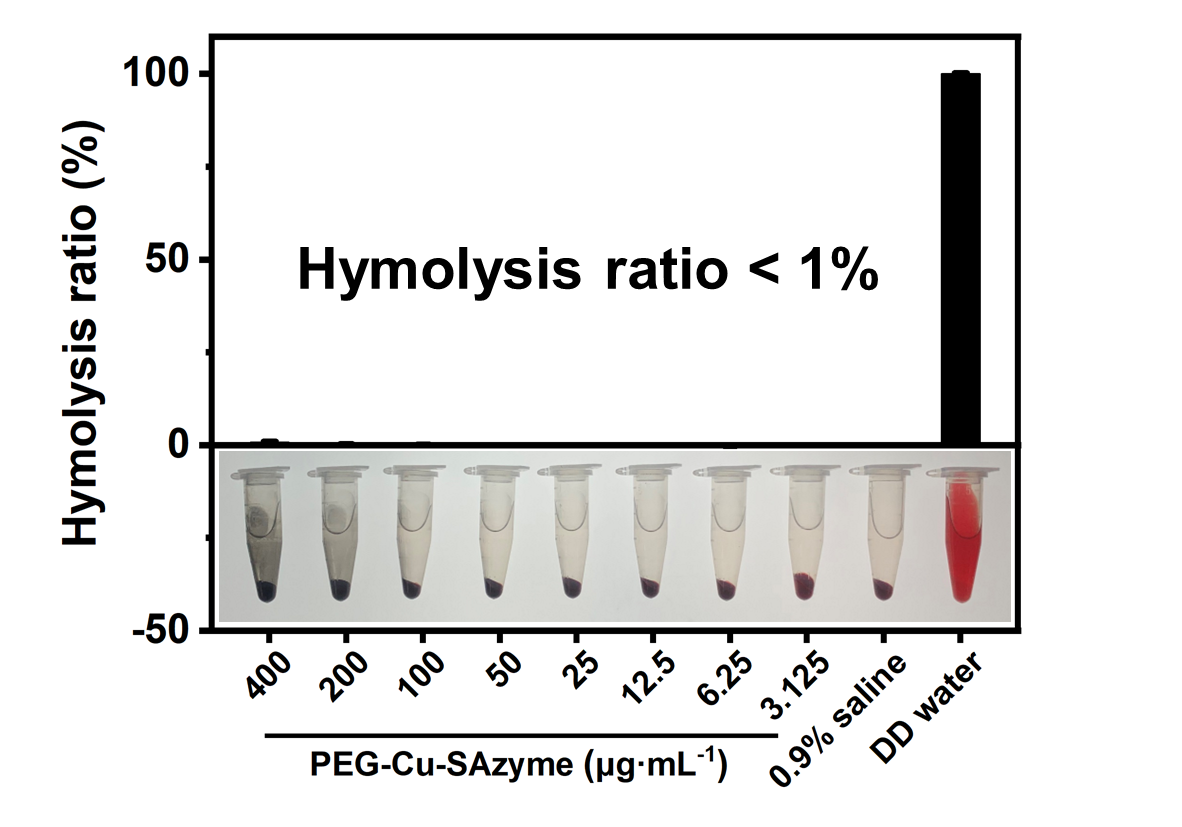


**Supplementary Figure 16.** Analysis of hemolysis characteristics of PEG-Cu-SAzyme. Inset: Photos of hemolysis in different systems.


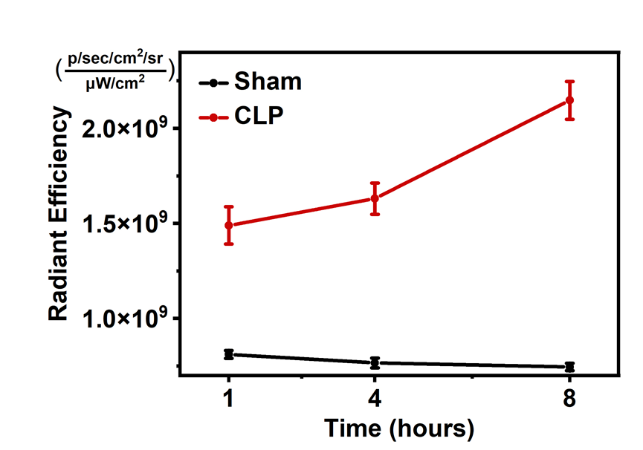


**Supplementary Figure 17.** Quantitative analysis of *in vivo* near-infrared fluorescence optical images obtained 1, 4, and 8 hours after Cy5.5-conjugated PEG-Cu-SAzyme was injected into CLP or Sham model (n= 3 per group).

**Supplementary Tables.**

**Supplementary Tables 1.** The element analysis of N-C and Cu-SAzyme resulted from XPS measurements.

| SAzyme | C at.% | O at.% | N at.% | Cu at.% | Pyridinic  N (%) | Pyrrolic  N (%) | Graphitic  N (%) | Oxidized  N (%) | N-O (%) | N-Cu  (%) |
| --- | --- | --- | --- | --- | --- | --- | --- | --- | --- | --- |
| N-C | 85.7 | 2.0 | 12.3 | / | 42 | 27.5 | 20.6 | 6.1 | 3.8 | / |
| Cu-SAzyme | 81.7 | 3.17 | 14.8 | 0.33 | 34 | 24.4 | 16.6 | 6.2 | 8.8 | 10 |

**Supplementary Tables 2.** The metal loading of all Cu-SAzymes determined by ICP-OES.

| SAzymes  (Theoretical loading) | N-C | 0.5wt% | 1.0wt% | 1.5wt% | 2.0wt% | CuNPs/NC |
| --- | --- | --- | --- | --- | --- | --- |
| ICP-OES | / | 0.45 | 0.91 | 1.34 | 2.00 | 11.50 |

**Supplementary Tables 3.** The structural parameters extracted from the Cu K-edge FT-EXAFS fitting.

| Sample | Shell | CN | R (Å) | σ^2^ (10^-2^Å^2^ ) | ΔE_0_ (eV) | r factor (%) |
| --- | --- | --- | --- | --- | --- | --- |
| Cu foil | Cu-Cu | 12 | 2.54 | - | - | - |
| Cu-SAzyme-fresh | Cu-N | 4.1 | 1.96 | 0.6 | -6.1 | 0.02 |
| Cu-SAzyme-used | Cu-N | 4.2 | 1.94 | 0.6 | -6.7 | 0.2 |

CN is the coordination number. R represents the interatomic distance. σ^2^ is the Debye-Waller factor. ΔE_0_ indicates the edge-energy shift. R factor is employed to assess the goodness of the fitting results.

**Supplementary Tables 4.** The comparisons of SOD-like activity between Cu-SAzyme and the others nanozymes reported recently.

| **Nanozymes/Enzymes** | **SOD activity (U·mg^-1^)** | **Detection system** | **Ref.** |
| --- | --- | --- | --- |
| Au/NC-3 | 4.43 | NBT | ^[5]^ |
| Fe_3_O_4_ | 5.65 | WST-1 | ^[6]^ |
| BMNPs | 21.88 | Pyrogallol autoxidation | ^[7]^ |
| VB_2_-IONzymes | 22.7 | WST-1 | ^[8]^ |
| CuNCs | 25.6 | WST-1 | ^[9]^ |
| Ferritin | 125 | WST-1 | ^[10]^ |
| CP-Pt | 142 | WST-1 | ^[11]^ |
| pero-nanozysome | 1257.1 | WST-1 | ^[12]^ |
| Natural SOD | 3091.8 | WST-1 | ^[12]^ |
| Cu-SAzyme | 448.22 | WST-1 | This Work |

**References**

[1] D. Rittirsch, M. S. Huber-Lang, M. A. Flierl, P. A. Ward, *Nat. Protoc.* **2009**, 4, 31.

[2] S. Suzuki, L. Toledo-Pereyra, F. Rodriguez, D. Cejalvo, *Transplantation* **1993**, 55, 1265.

[3] M. S. Paller, J. Hoidal, T. F. Ferris, *J. Clin. Invest.* **1984**, 74, 1156.

[4] Y. Hirano, M. Aziz, W.-L. Yang, Z. Wang, M. Zhou, M. Ochani, A. Khader, P. Wang, *Crit. Care* **2015**, 19, 53.

[5] J. Zhao, H. Wang, H. Geng, Q. Yang, Y. Tong, W. He, *ACS Appl. Nano Mater.* **2021**, 4, 7253.

[6] B. C. Yan, J. Cao, J. Liu, Y. Gu, Z. Xu, D. Li, L. Gao, *ACS Biomater. Sci. Eng.* **2020**, 7, 299.

[7] Y. Pan, Y. Wang, X. Fan, W. Wang, X. Yang, D. Cui, M. Zhao, *Environ. Microbiol. Rep.* **2019**, 11, 140.

[8] Y. Gu, Y. Huang, Z. Qiu, Z. Xu, D. Li, L. Chen, J. Jiang, L. Gao, *Sci. China: Life Sci.* **2020**, 63, 68.

[9] Y. Peng, Y. Ren, H. Zhu, Y. An, B. Chang, T. Sun, *RSC Adv.* **2021**, 11, 14517.

[10] J. Zhang, S. Li, N. Xie, G. Nie, A. Tang, X.-E. Zhang, M. Liang, X. Yan, *Sci. China: Life Sci.* **2021**, 64, 1375.

[11] M. Kajita, K. Hikosaka, M. Iitsuka, A. Kanayama, N. Toshima, Y. Miyamoto, *Free Radical Res.* **2007**, 41, 615.

[12] J. Xi, R. Zhang, L. Wang, W. Xu, Q. Liang, J. Li, J. Jiang, Y. Yang, X. Yan, K. Fan, *Adv. Funct. Mater.* **2021**, 31, 2007130.
